# Author Correction: Cytoplasmic Endonuclease G promotes nonalcoholic fatty liver disease via mTORC2-AKT-ACLY and endoplasmic reticulum stress

**DOI:** 10.1038/s41467-024-51312-x

**Published:** 2024-08-21

**Authors:** Wenjun Wang, Junyang Tan, Xiaomin Liu, Wenqi Guo, Mengmeng Li, Xinjie Liu, Yanyan Liu, Wenyu Dai, Liubing Hu, Yimin Wang, Qiuxia Lu, Wen Xing Lee, Hong-Wen Tang, Qinghua Zhou

**Affiliations:** 1 The Sixth Affiliated Hospital of Jinan University (Dongguan Eastern Central Hospital), Jinan University, Dongguan, Guangdong 523067 China; 2 The Biomedical Translational Research Institute, Health Science Center (School of Medicine), Jinan University, Guangzhou, Guangdong 510632 China; 3 The First Affiliated Hospital, Jinan University, Guangzhou, Guangdong 510632 China; 4 GeneMind Biosciences Company Limited, No. 116, Qingshuihe 1st Road, Luohu District, Shenzhen, Guangdong 518000 China; 5 School of Food and Biological Engineering, Chengdu University, Chengdu, 610106 China; 6 Program in Cancer and Stem Cell Biology, Duke-NUS Medical School, 8 College Road, Singapore, 169857 Singapore; 7 Division of Cellular & Molecular Research, Humphrey Oei Institute of Cancer Research, National Cancer Centre Singapore, Singapore, 169610 Singapore

Correction to: *Nature Communications* 10.1038/s41467-023-41757-x, published online 04 October 2023

The original version of this Article contained an error in Fig. 1 and in its associated Results and Methods sections, where it was inadvertently omitted that the liver tissue examined in panels a–f came from mice that were starved for 24 h. This has been corrected in both the PDF and HTML versions of the Article.

