# Supplementary material for: Cytoplasmic Endonuclease G promotes nonalcoholic fatty liver disease via mTORC2-AKT-ACLY and endoplasmic reticulum stress
Source: Nat Commun. 2023 Oct 4;14:6201. doi: 10.1038/s41467-023-41757-x (PMC10550995; doi:10.1038/s41467-023-41757-x)
Supplement: Supplementary file 1 — Supplementary Information [file 41467_2023_41757_MOESM1_ESM.pdf]

Supplementary Information for

Cytoplasmic Endonuclease G promotes nonalcoholic fatty liver disease via mTORC2-AKT-ACLY and endoplasmic reticulum stress

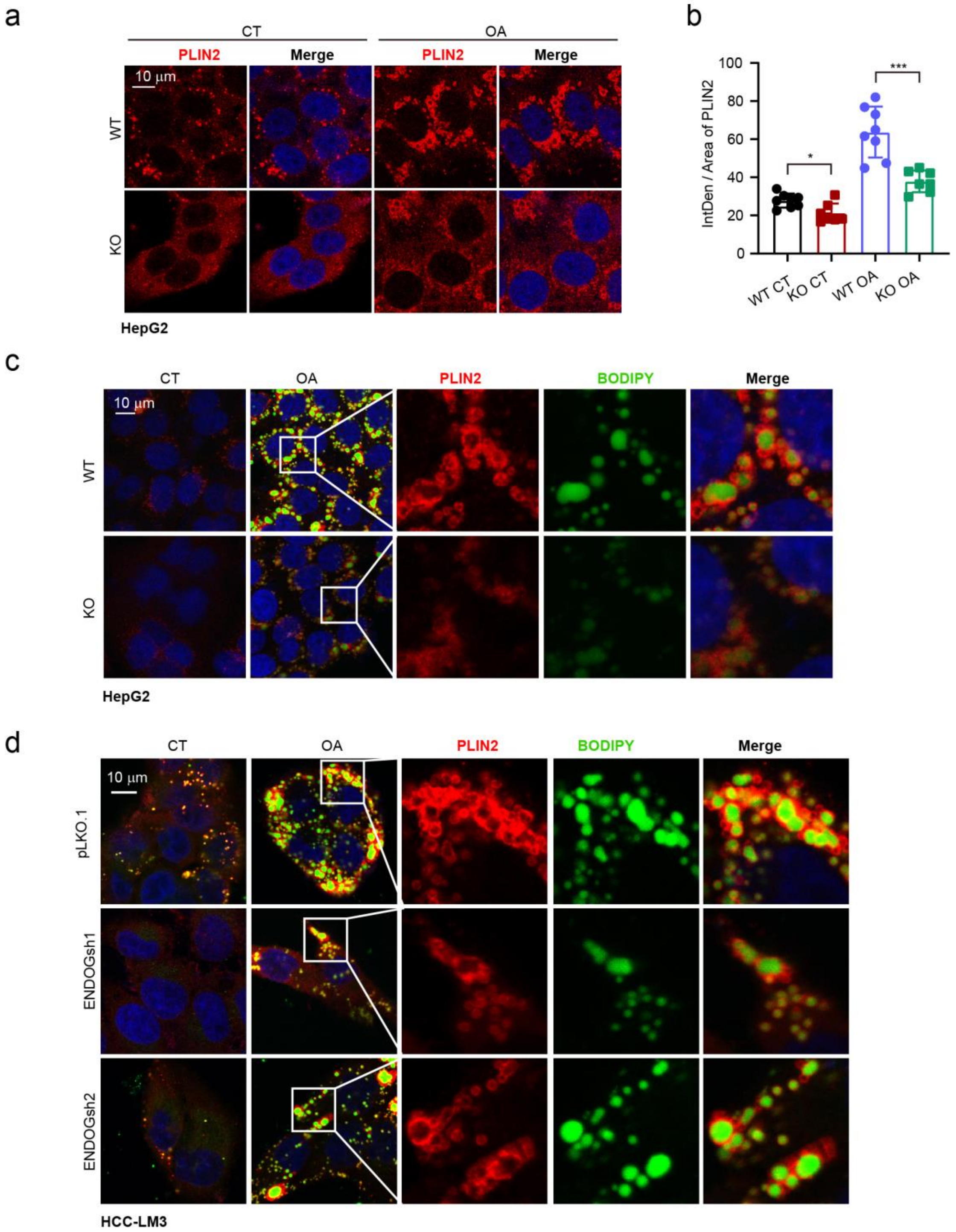

Figure S1. Loss of ENDOG represses PLIN2 expression and lipid accumulation in hepatocytes. a-b Representative images and quantitative

results of PLIN2 staining in HepG2 cells under control and oleic acid treatment. CT: control; OA: 200 μM oleic acid for 24 hours; *n* = 8 samples; WT: wild-type, KO: ENDOG knockout. **c-d** Representative images of BODIPY/ PLIN2 co-staining in HepG2 and HCC-LM3 cells. CT: control; OA: 200 μM oleic acid for 24 hours; WT: wild-type, KO: ENDOG knockout. Statistical significance was determined by unpaired Student's t-test (two-tailed) in **(b)**; error bars are mean ± SD. Source data and exact *P* values are provided in a Source data file. * $P < 0.05$; *** $P < 0.001$

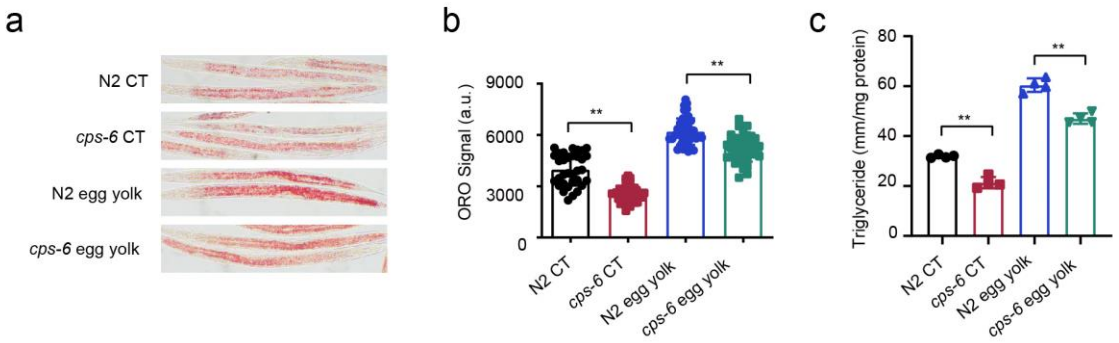

Figure S2. Loss of ENDOG represses lipid accumulation in *C. elegans* after egg yolk supplementation. a-b Representative images and quantitative results of oil red o staining in N2 and *cps-6* mutant *C. elegans*. N2: wild-type; *cps-6*: loss of CPS-6; CT: control; egg yolk: egg yolk supplementation; *n* = 30 *C. elegans*. **c** Whole measurement of triglycerides. *n* = 4 independent samples; N2: wild-type; cps-6: loss of CPS-6; CT: control; egg yolk: egg yolk supplementation. Statistical significance was determined by unpaired Student's t-test (two-tailed) in **(b, c)**; error bars are mean ± SD. Source data and exact *P* values are provided in a Source data file. ** $P < 0.01$.

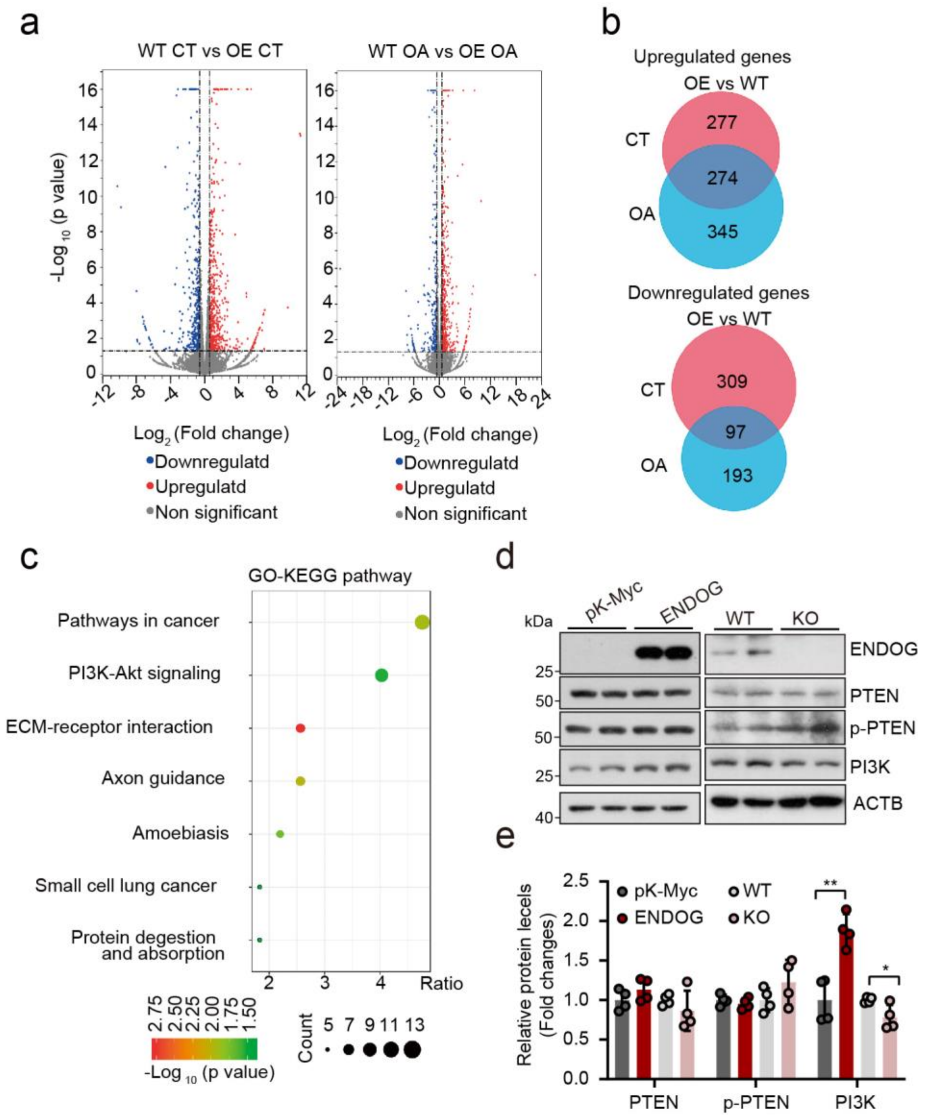

Figure S3. ENDOG promotes the activation of AKT. a Volcano plots of gene expression differences for wild-type or ENDOG overexpressing cells in control or oleic acid treatment. **b** Venn diagram of up-regulated (up panel) or down-regulated (below panel) between wild-type and ENDOG overexpressing cells. **c** The bubble map of KEGG pathway enrichment analyses of the up-regulated genes. **d-e** Representative western blots (d) and the quantitative results (e) of the indicated proteins in ENDOG overexpressing and knockout cells. pK-Myc and ENDOG plasmids were transfected for 48 hours, $n = 4$ each group; WT: wild-type; KO: ENDOG knockout. Statistical significance was determined by unpaired Student's t-test (two-tailed) in **(e)**; error bars are mean ± SD. Source data and exact P values are provided in a Source data file. * $P < 0.05$; ** $P < 0.01$.

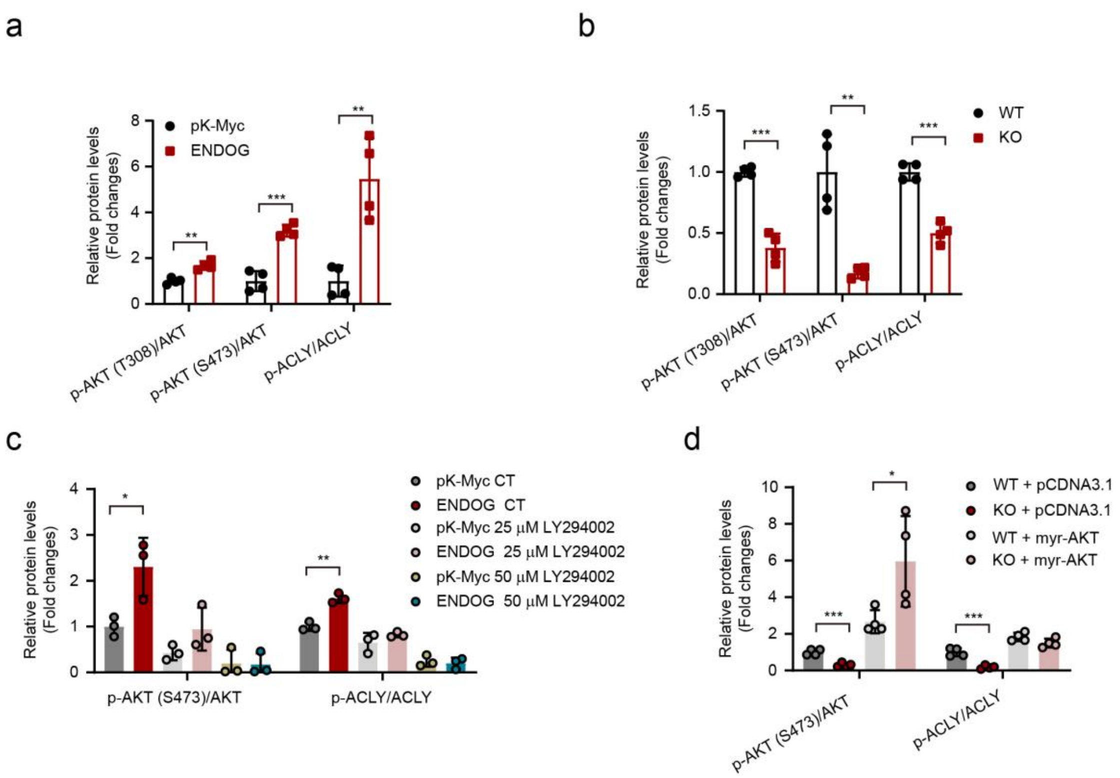

Figure S4. ENDOG promoted the phosphorylation of AKT and ACLY. a-b Quantitative results of western blots in Figure 2 a-b. *n* = 4 samples. **c** Quantitative results of western blots in Figure 2 k. *n* = 4 samples. **d** Quantitative results of western blots in Figure 2 o. *n* = 4 samples; Statistical significance was determined by unpaired Student's t-test (two-tailed). Source data and exact *P* values are provided in a Source data file. * $P < 0.05$; ** $P < 0.01$; *** $P < 0.001$.

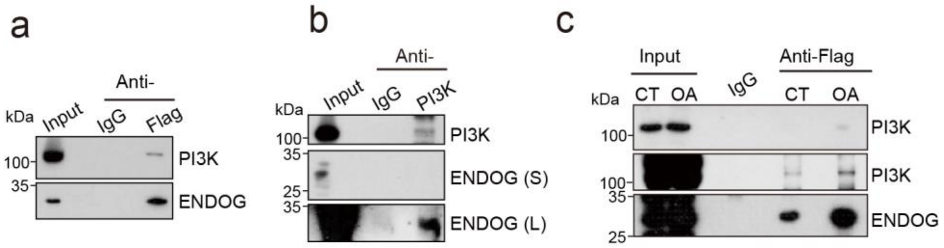

Figure S5. The oleic acid treatment enhances the binding between ENDOG and PI3K. a Co-IP analyses. Cells were transfected with ENDOG-Flag for 48 hours. **b** Endogenous Co-IP analyses. **C** Co-IP analyses. Cells were transfected with ENDOG-Flag for 24 hours and then treated with 200 μM oleic acid for another 24 hours. These data had three independent experiments with similar results.

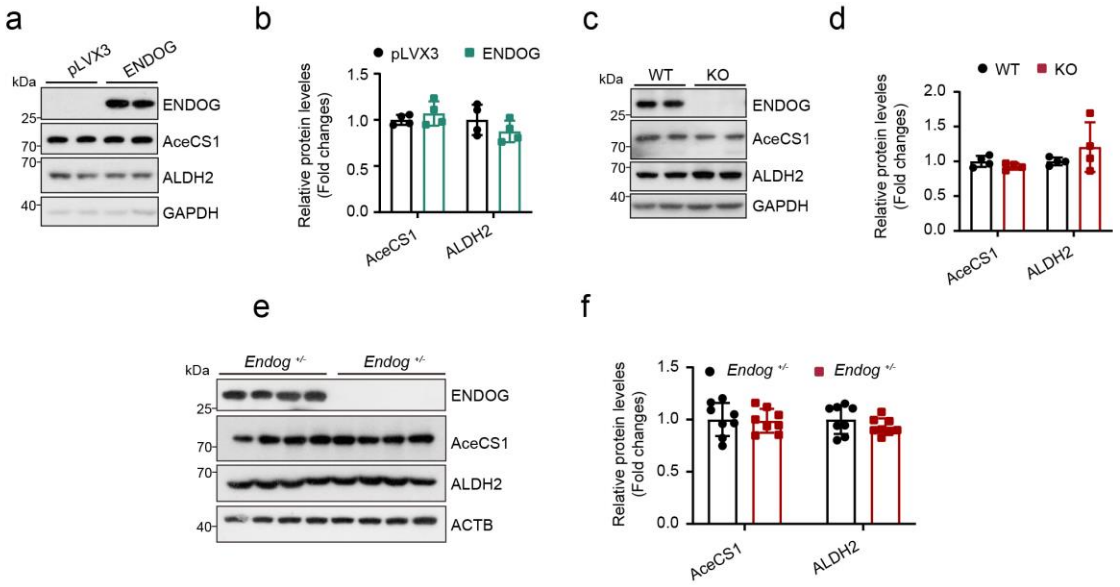

Figure S6. ENDOG did not affect the acetate metabolism enzymes. a-b Representative western blots (a) and the quantitative results (b) of AceCS1 and ALDH2 in ENDOG overexpressed HepG2 cells. *n* = 4 samples; pLVX3: the control HepG2; ENDOG : ENDOG stable overexpressed HepG2. **c-d** Representative western blots (c) and the quantitative results (d) of AceCS1 and ALDH2 in wild-type and ENDOG knockout HepG2 cells. *n* = 4 samples; WT: wild-type; KO: ENDOG knockout. **e-f** Representative western blots (c) and the quantitative results (d) of AceCS1 and ALDH2 in Endog$^{+/-}$ and Endog$^{-/-}$ female mice livers. *n* = 8 mice. Statistical significance was determined by unpaired Student's t-test (two-tailed) in **(b,d,f)**; error bars are mean ± SD.Source data and exact *P* values are provided in a Source data file.

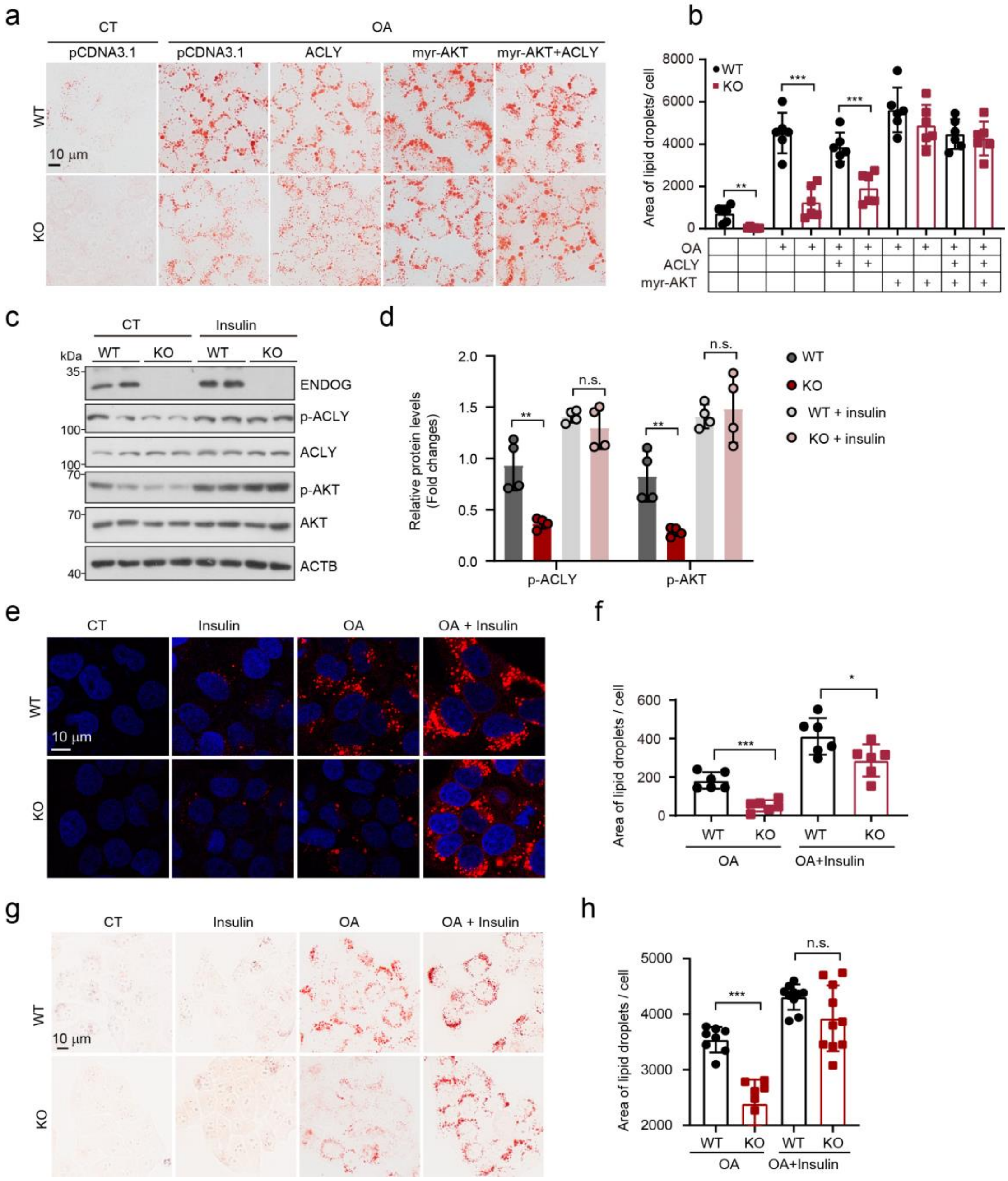

Figure S7. Activation of AKT-ACLY signaling repromotes lipid accumulation in ENDOG knockout cells. a-b Representative images of oil red O staining and the quantitative results of lipid area in the indicated groups. Wild-type and ENDOG knockout cells were transfected with the indicated plasmids for 24 hours and then treated with 200 μM oleic acid for another 24 hours. n = 6 samples. **c-d** Western blot analyses and the quantitative results of AKT-ACLY signaling in wild-type and ENDOG knockout cells following 100 nM insulin treatment for 6 hours. n = 4 samples. **e-h** Representative images of Nile

red (e-f) or oil red O (g-h) staining and the related quantitative results of lipid area in wild-type or ENDOG knockout cells after the indicated treatments. *n* = 6 for Nile red staining and 10 for oil o staining; Insulin, 100 nM for 6 hours, OA: 200 μM for 24 hours. OA + insulin: 100 nM insulin for 6 hours and then replaced with 200 μM oleic acid-contained media for another 24 hours. Statistical significance was determined by unpaired Student's t-test (two-tailed) in **(b,d,f,h)**; error bars are mean ± SD.Source data and exact *P* values are provided in a Source data file. * $P < 0.05$; ** $P < 0.01$, *** $P < 0.001$, n.s.: no significance.

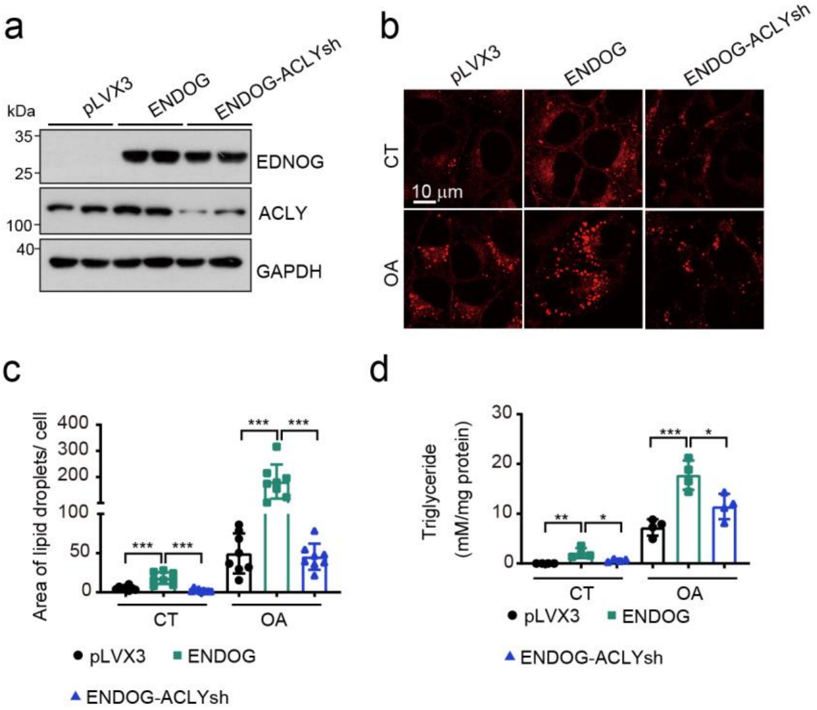

Figure S8. Knockdown of ACLY repressed ENDOG-induced lipid accumulation. a Western blot of ENDOG and ACLY. pLVX3: the control cells; ENDOG: ENDOG overexpressing cells; ENDOG-ACLYsh: ENDOG overexpressing and ACLY knockdown. **b-d** Representative images of Nile red, the quantitative results of lipid area, and measurement of triglycerides following 200 μM oleic acid treatment for 24 hours. *n* = 8 for Nile red staining and 4 for triglyceride measurement. Statistical significance was determined by unpaired

Student's t-test (two-tailed) in **(c,d,)**; error bars are mean ± SD. Source data and exact *P* values are provided in a Source data file. * $P < 0.05$; ** $P < 0.01$, *** $P < 0.001$.

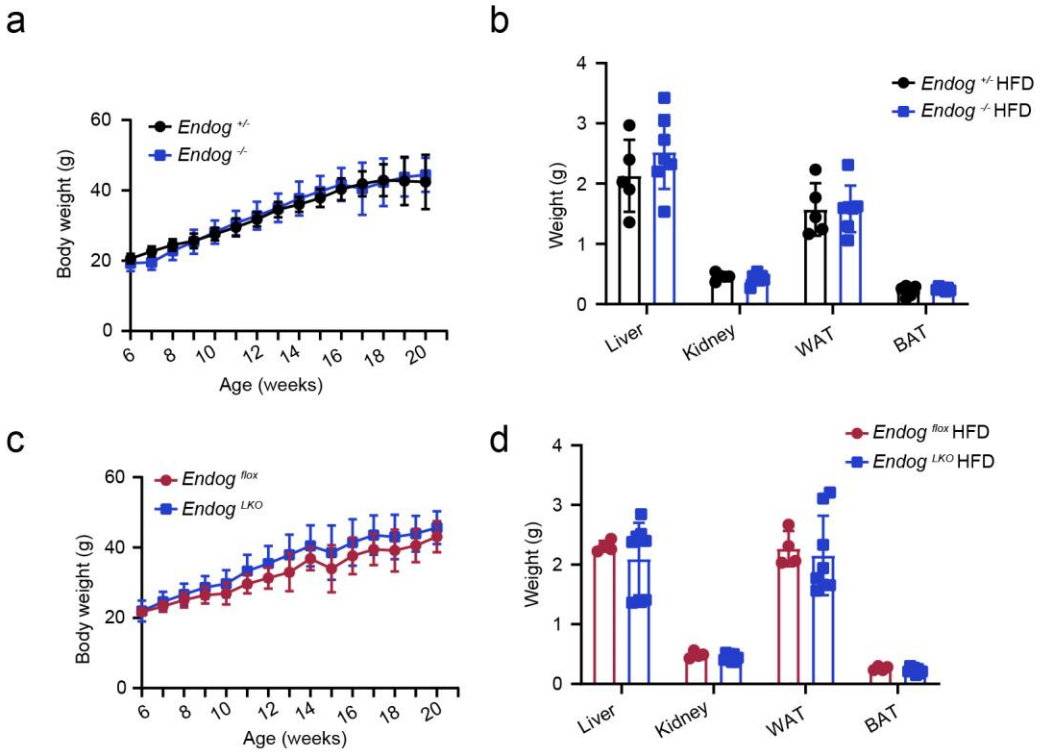

Figure S9. Loss of ENDOG had no effects on the body weight and NFBG male mice after HFD. a, c Body weight of ENDOG knockout (a) and liver-specific knockout male mice (b) after HFD chow. *n* = 5 in *Endog* $^{+/-}$ group and 7 in *Endog*$^{-/-}$ group. **b, d** Weight of liver, kidney, white adipose tissue (WAT), and brown adipose tissue (BAT). *n* = 5 in *Endog* $^{+/-}$ group and 7 in *Endog*$^{-/-}$ group. Statistical significance was determined by unpaired Student's t-test (two-tailed); error bars are mean ± SD. Source data and exact *P* values are provided in a Source data file.

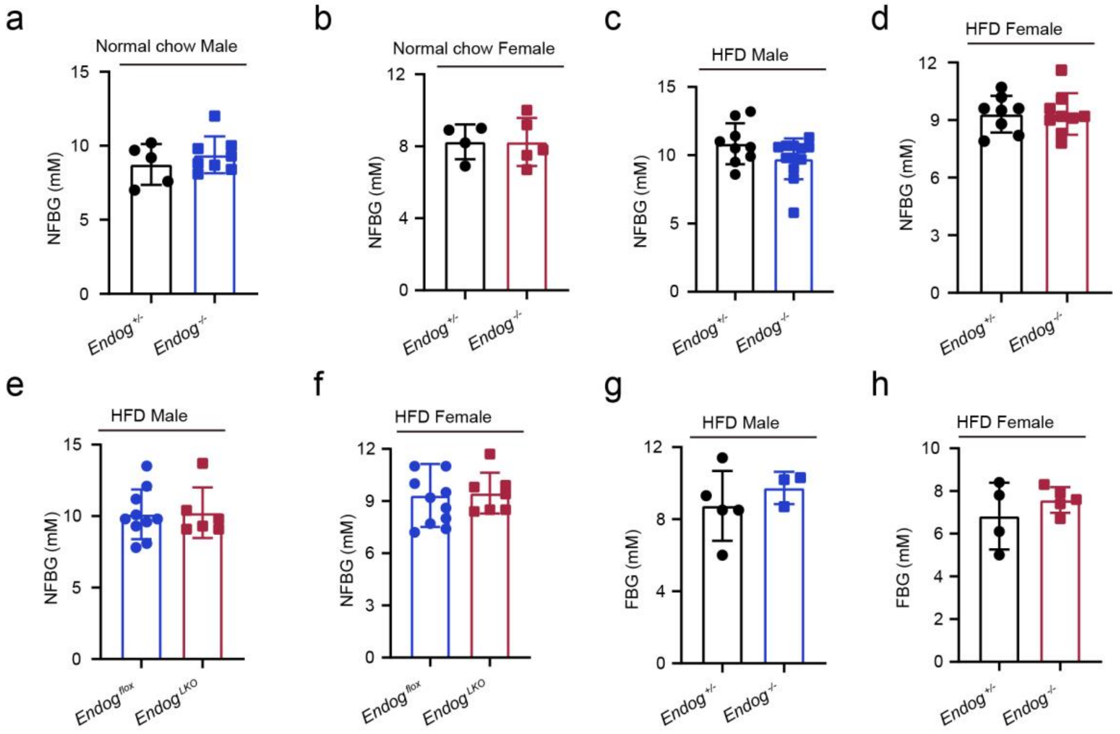

Figure S10. Loss of ENDOG had no influence on the blood glucose both in normal and HFD chow. a-b Nonfasting blood glucose (NFBG) in the normal chow condition. n = 5, 8 in *Endog*$^{+/-}$ / *Endog*$^{-/-}$male; *n* = 4, 5 in *Endog*$^{+/-}$ / *Endog*$^{-/-}$ female. **c-d** Nonfasting blood glucose (NFBG) in the HFD chow condition. *n* = 9, 12 in *Endog*$^{+/-}$ / *Endog*$^{-/-}$male; *n* = 8, 9 in *Endog*$^{+/-}$ / *Endog*$^{-/-}$ female. **e-f** Nonfasting blood glucose (NFBG) in *Endog*^{flox} / *Endog*^{LKO} male and female mice under the HFD condition. *n* = 6, 10 in *Endog*^{flox} / *Endog*^{LKO} male; *n* = 11, 7 in *Endog*^{flox} / *Endog*^{LKO} female. **g-h** Fasting blood glucose (FBG) in Endog$^{+/-}$ / Endog$^{-/-}$ male and female mice under the HFD chow condition. Before blood glucose measurement, mice were fasting for 12 hours. *n* = 5, 3 in *Endog*$^{+/-}$ / *Endog*$^{-/-}$male; *n* = 4, 5 in *Endog*$^{+/-}$ / *Endog*$^{-/-}$ female. Statistical significance was determined by unpaired Student's t-test (two-tailed); error bars are mean ± SD. Source data and exact *P* values are provided in a Source data file.

a

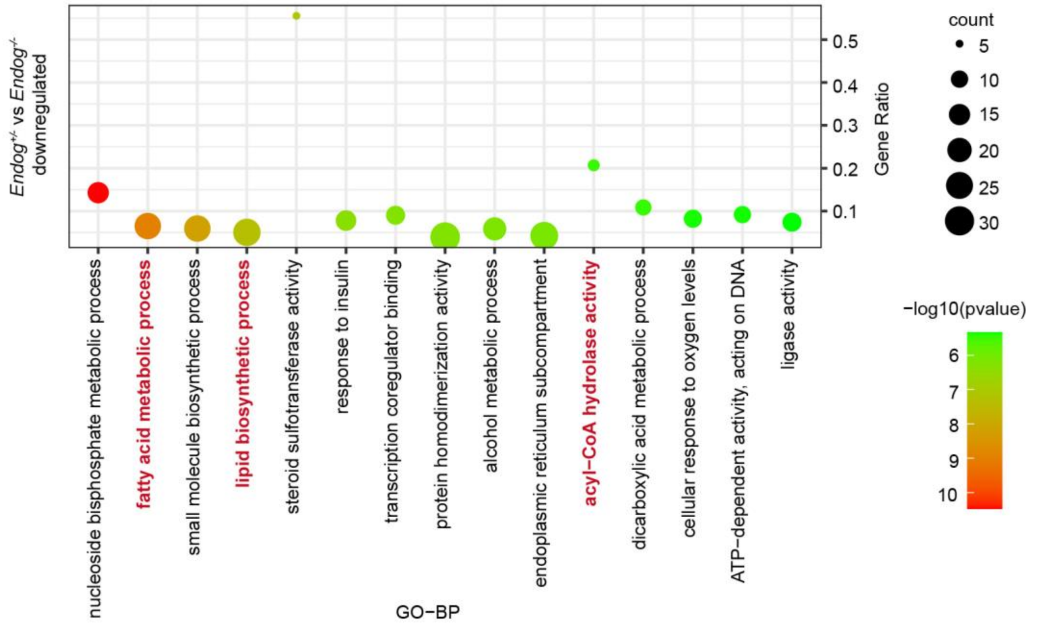

b

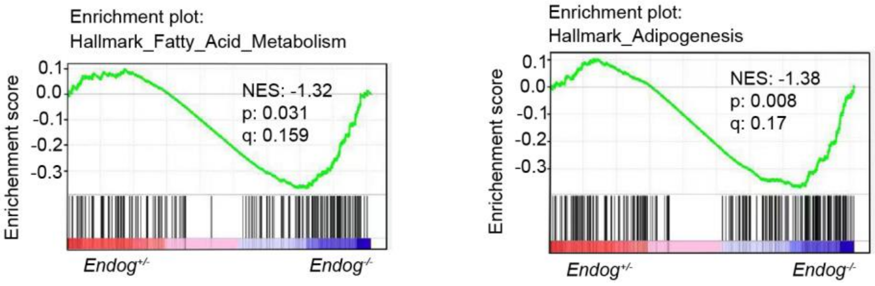

Figure S11. Loss of ENDOG represses the lipid metabolism pathway. a The bubble map of KEGG pathway enrichment analyses of the down-regulated genes in *Endog* $^{+/-}$ and *Endog* $^{-/-}$ mouse livers following the HFD chow feeding. **b** GSEA (Gene Set Enrichment Analyses) shows the biological processes enriched in *Endog* $^{+/-}$ liver. (NES: normalized enrichment score; p: nominal p-value; q: false discovery rate q-value).

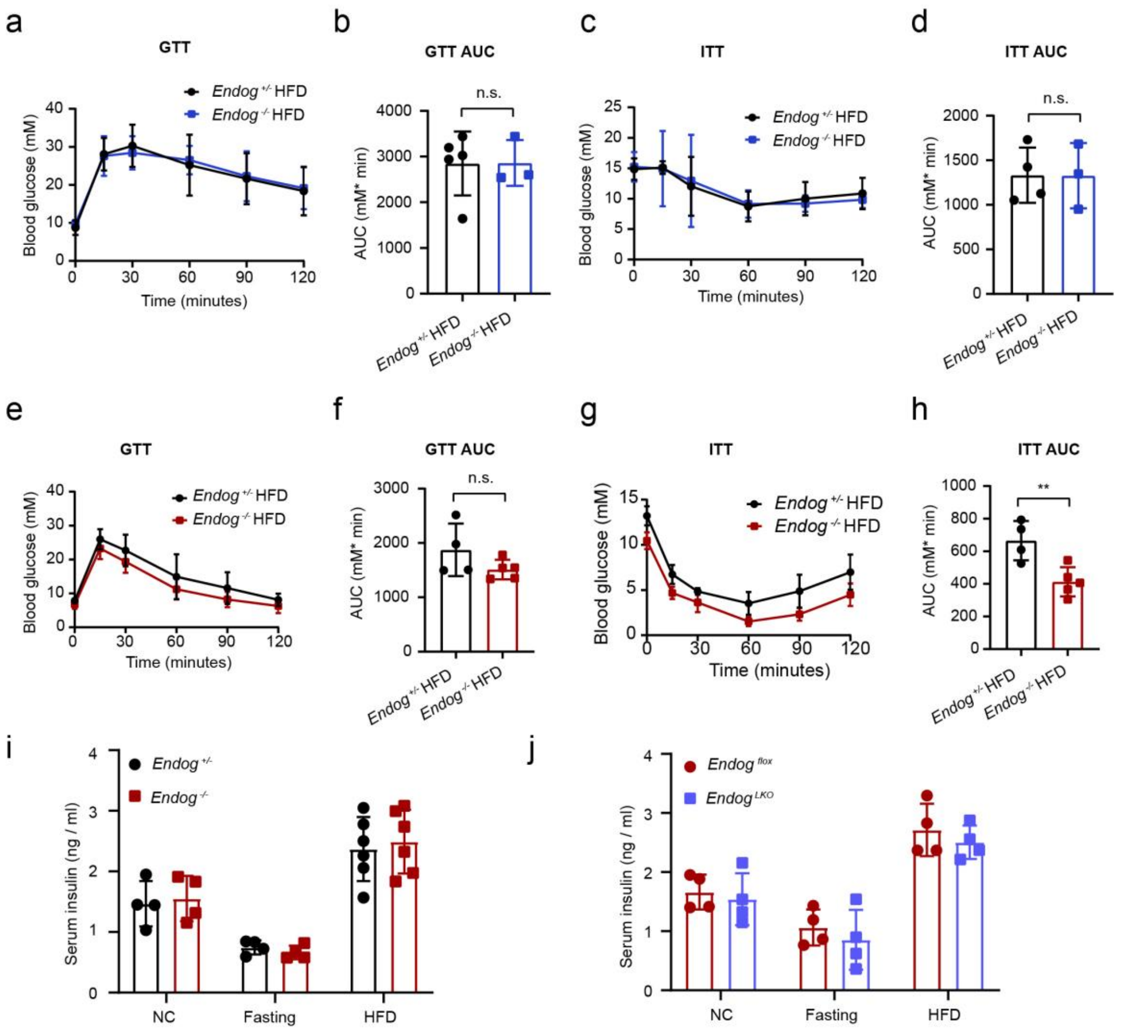

Figure S12. Loss of ENDOG increased insulin sensitivity in female mice after the HFD chow. a-d Glucose tolerance test (a-b) and insulin tolerance test (c-d) in HFD-fed Endog$^{+/-}$ / Endog$^{-/-}$ male mice. *n* = 5, 3 for GTT assay; *n* = 4,3 for ITT assay. **e-h** Glucose tolerance test (a-b) and insulin tolerance test (c-d) in HFD-fed *Endog*$^{+/-}$ / *Endog*$^{-/-}$ female mice. *n* = 4, 5. **i-j** Serum insulin in ENDOG knockout and liver-specific knockout female mice in normal, fasting, and HFD conditions. *n* = 4 for NC and Fasting insulin measurement and HFD insulin measurement in liver-specific knockout mice; *n* = 6 for HFD insulin measurement in ENDOG knockout mice. NC: normal chow; fasting: fasting for 16 hours; HFD: high-fat diet chow for 16 weeks. Statistical significance was determined by unpaired Student's t-test (two-tailed); error bars are mean ± SD. Source data and exact *P* values are provided in a Source data file. n.s.: no significance; ** *P* < 0.01.

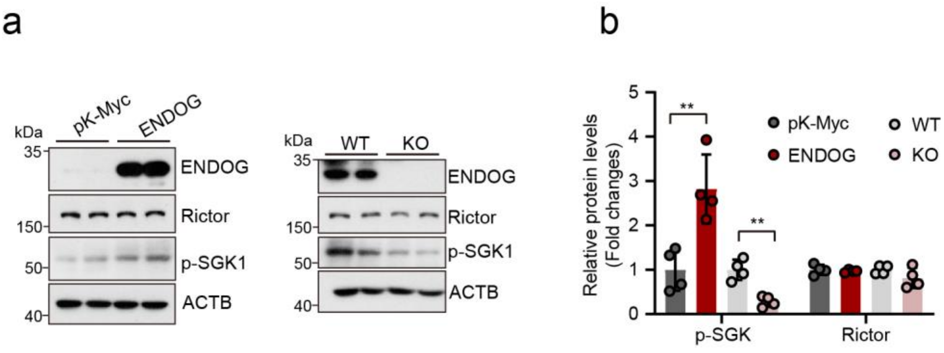

Figure S13. ENDOG promotes the phosphorylation of SGK1. a-b Western blots and the quantitative results of Rictor and phosphorylation of SGK1 in ENDOG overexpressing and knockout cells. Cells were transfected with pK-Myc or ENDOG plasmids for 48 hours. *n* = 4. WT: wild-type, KO: ENDOG knockout. Statistical significance was determined by unpaired Student's t-test (two-tailed); error bars are mean ± SD. Source data and exact *P* values are provided in a Source data file. * *P* < 0.05; ** *P* < 0.01.

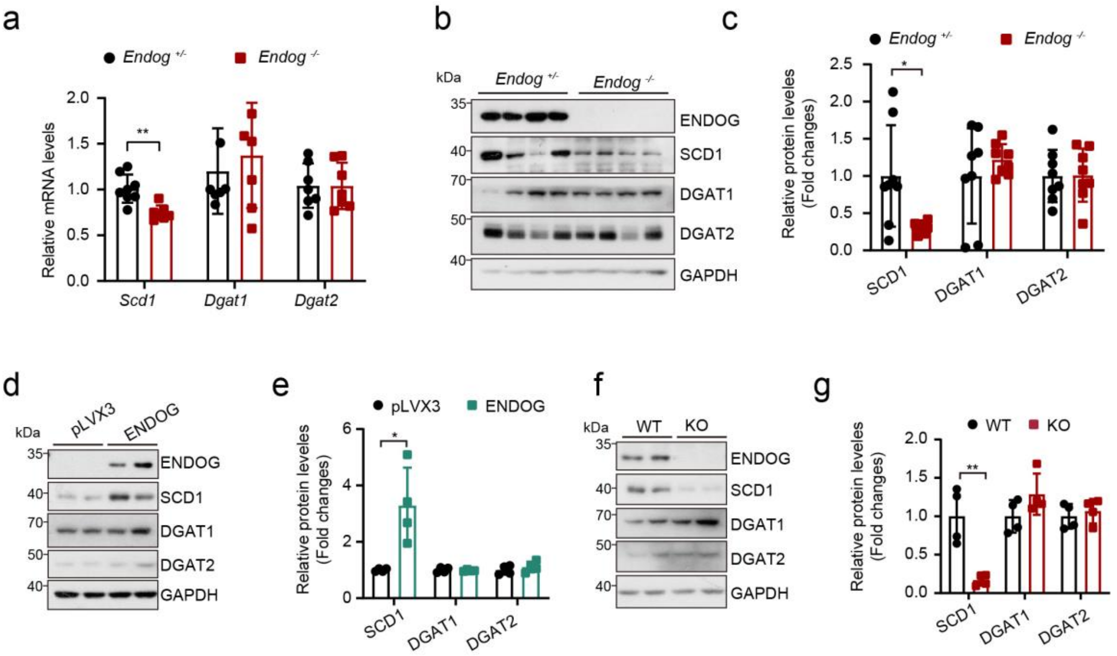

Figure S14. ENDOG promoted the expression of SCD1. a-c qPCR results (a), representative western blots (b), and the quantitative results (c) of SCD1, DGAT1, and DGAT2 in Endog$^{+/-}$ / Endog$^{-/-}$ female mice liver. *n* = 6 for each group. **d-g** Representative western blots and the quantitative results of SCD1, DGAT1, and DGAT2 in ENDOG overexpressed (d-e) and knockout (f-g) HepG2 cells.

pLVX3: the control HepG2; ENDOG: ENDOG overexpressed HepG2; *n* = 4 each group. WT: wild-type; KO: ENDOG knockout; Statistical significance was determined by unpaired Student's t-test (two-tailed); error bars are mean ± SD. Source data and exact *P* values are provided in a Source data file. * $P < 0.05$; ** $P < 0.01$.

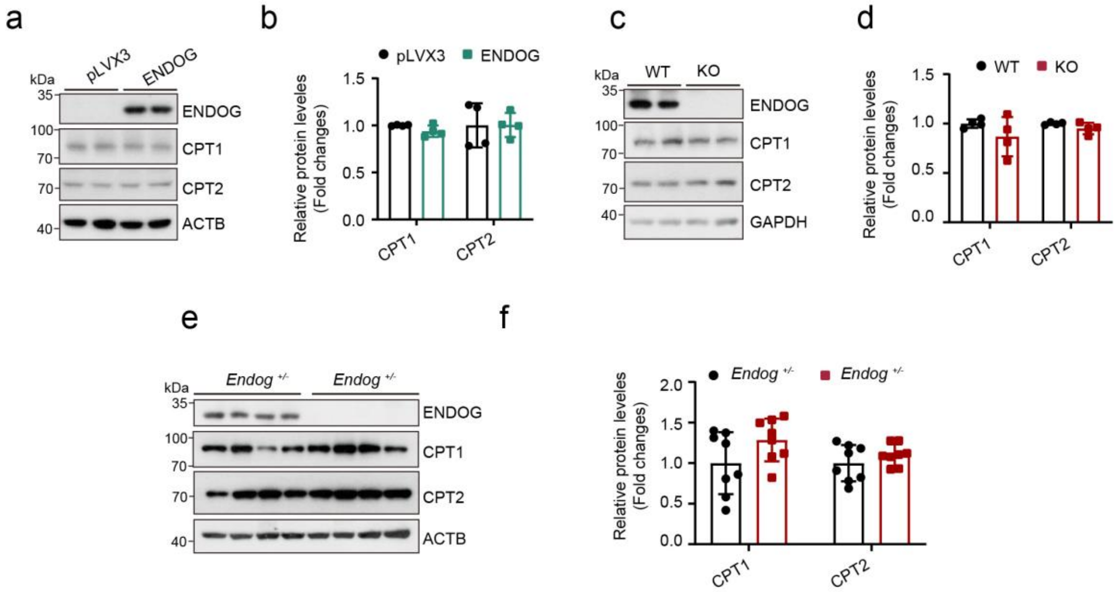

Figure S15. Overexpression or knockout of ENDOG had no effects on fatty acid β-oxidation. a-d Representative western blots and the quantitative results of CPT1 and CPT2 in ENDOG overexpressed (a-b) and knockout (c-d) HepG2 cells. *n* = 4 for each group. pLVX3: the control HepG2; ENDOG: ENDOG overexpressed HepG2; WT: wild-type; KO: ENDOG konockout. **e-f** Representative western blots and the quantitative results of CPT1 and CPT2 in Endog$^{+/-}$ / Endog$^{-/-}$ female mice liver. *n* = 8 for each group. Statistical significance was determined by unpaired Student's t-test (two-tailed); error bars are mean ± SD. Source data and exact *P* values are provided in a Source data file.

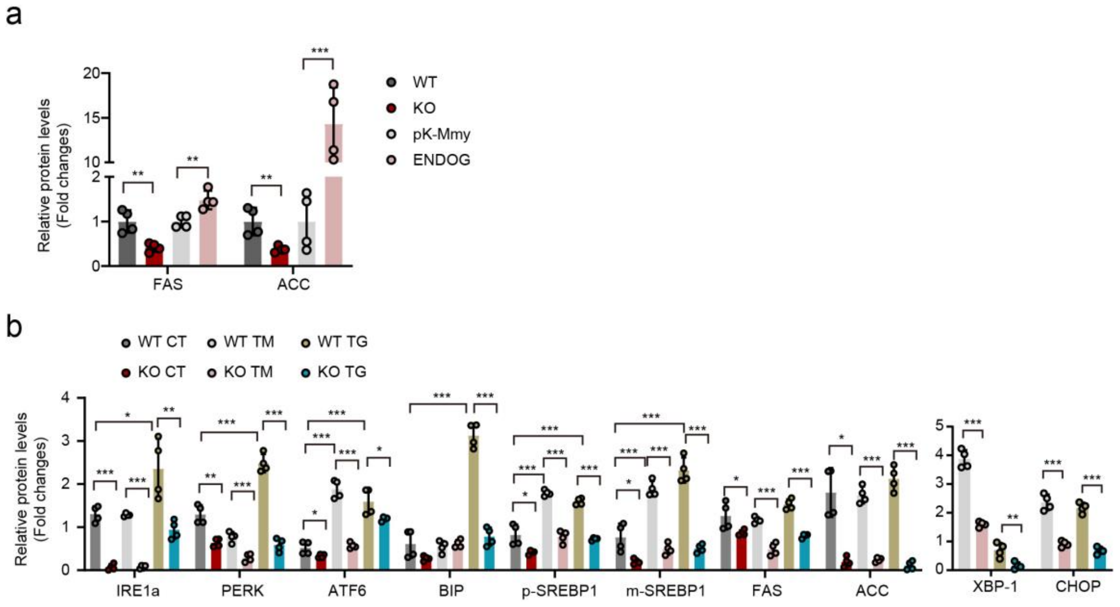

Figure S16. Loss of ENDOG repressed ER stress. a Quantitative results of western blots in Figure 5 b-c. *n* = 4 for each group. **b** Quantitative results of western blots in Figure 5 e. *n* = 4 for each group. Statistical significance was determined by unpaired Student's t-test (two-tailed); error bars are mean ± SD. Source data and exact *P* values are provided in a Source data file. * $P < 0.05$; ** $P < 0.01$; *** $P < 0.001$.

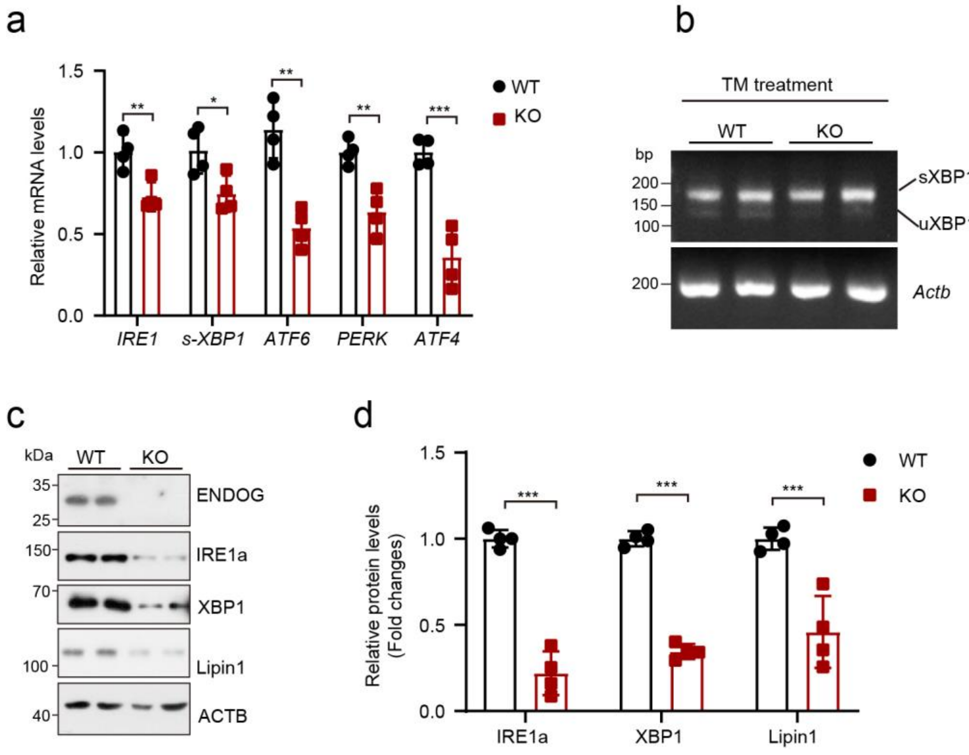

Figure 17. Loss of ENDOG represses IRE1a-XBP1 activation and Lipin1

expression. a qPCR results of the ER stress-related genes in wild-type and ENDOG knockout cells. *n* = 4 for each group. **b** RT-PCR analyses of *XBP1*. Wild-type and ENDOG knockout cells were treated with 2 μg/ml tunicamycin (TM) for 24 hours. **c-d** Western blots and the quantitative results of the indicated protein in wild-type and ENDOG knockout cells. *n* = 4 for each group. Statistical significance was determined by unpaired Student's t-test (two-tailed); error bars are mean ± SD. Source data and exact *P* values are provided in a Source data file. * *P* < 0.05; ** *P* < 0.01; *** *P* < 0.001.

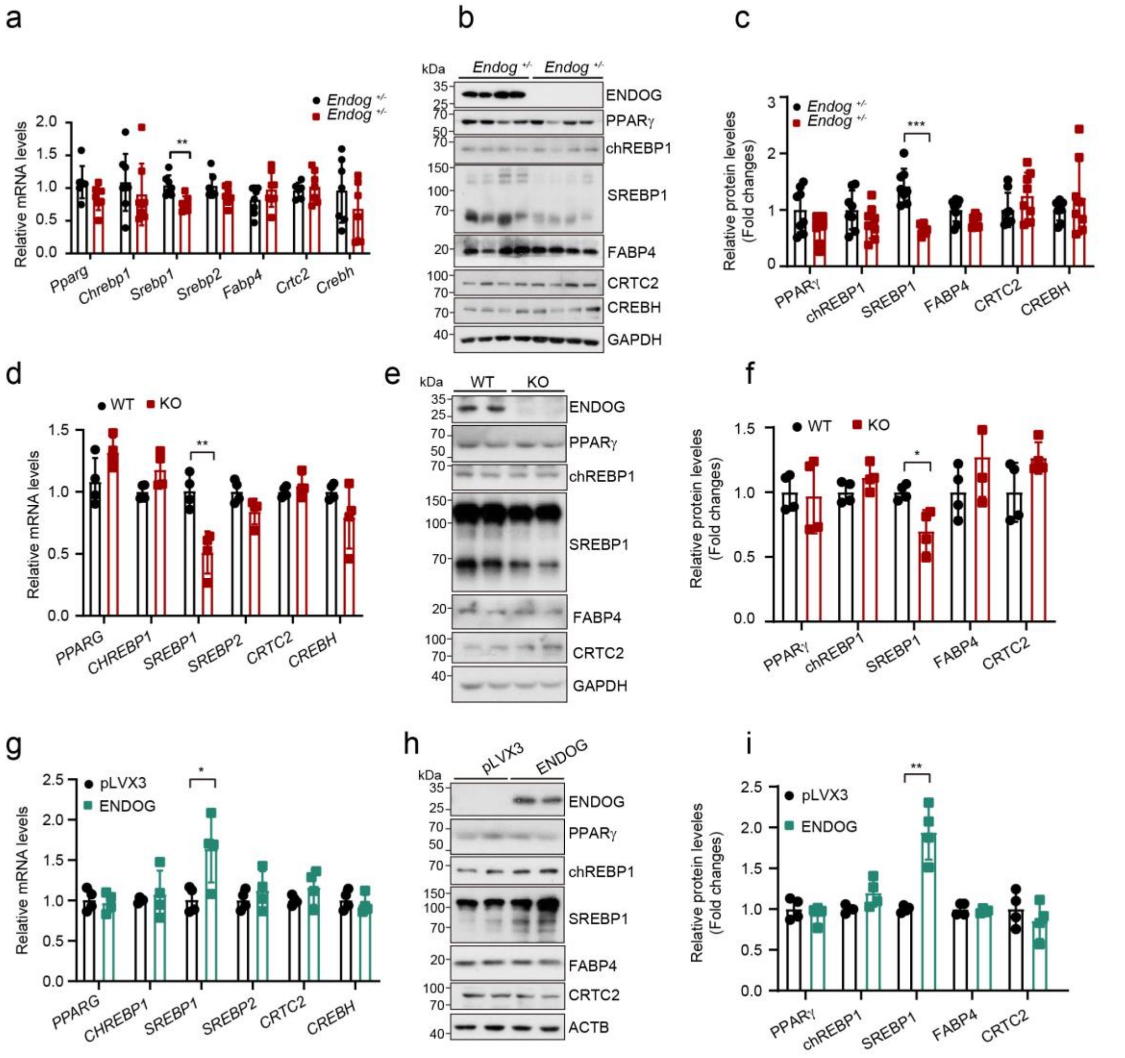

Figure S18. ENDOG promoted the expression of SREBP1 but not other lipid metabolism transcriptional factors. a-c qPCR results (a), representative western blots (b), and the quantitative results (c) of the indicated transcriptional factors in Endog$^{+/-}$ / Endog$^{-/-}$ female mice liver. *n* = 8 for each

group. **d-i** qPCR results, representative Western blots, and the quantitative results of the indicated transcriptional factors in ENDOG knockout (d-f) and overexpressed (g-i) HepG2. $n = 4$ for each group. Statistical significance was determined by unpaired Student's t-test (two-tailed); error bars are mean ± SD. Source data and exact P values are provided in a Source data file. * $P < 0.05$; ** $P < 0.01$; *** $P < 0.001$.

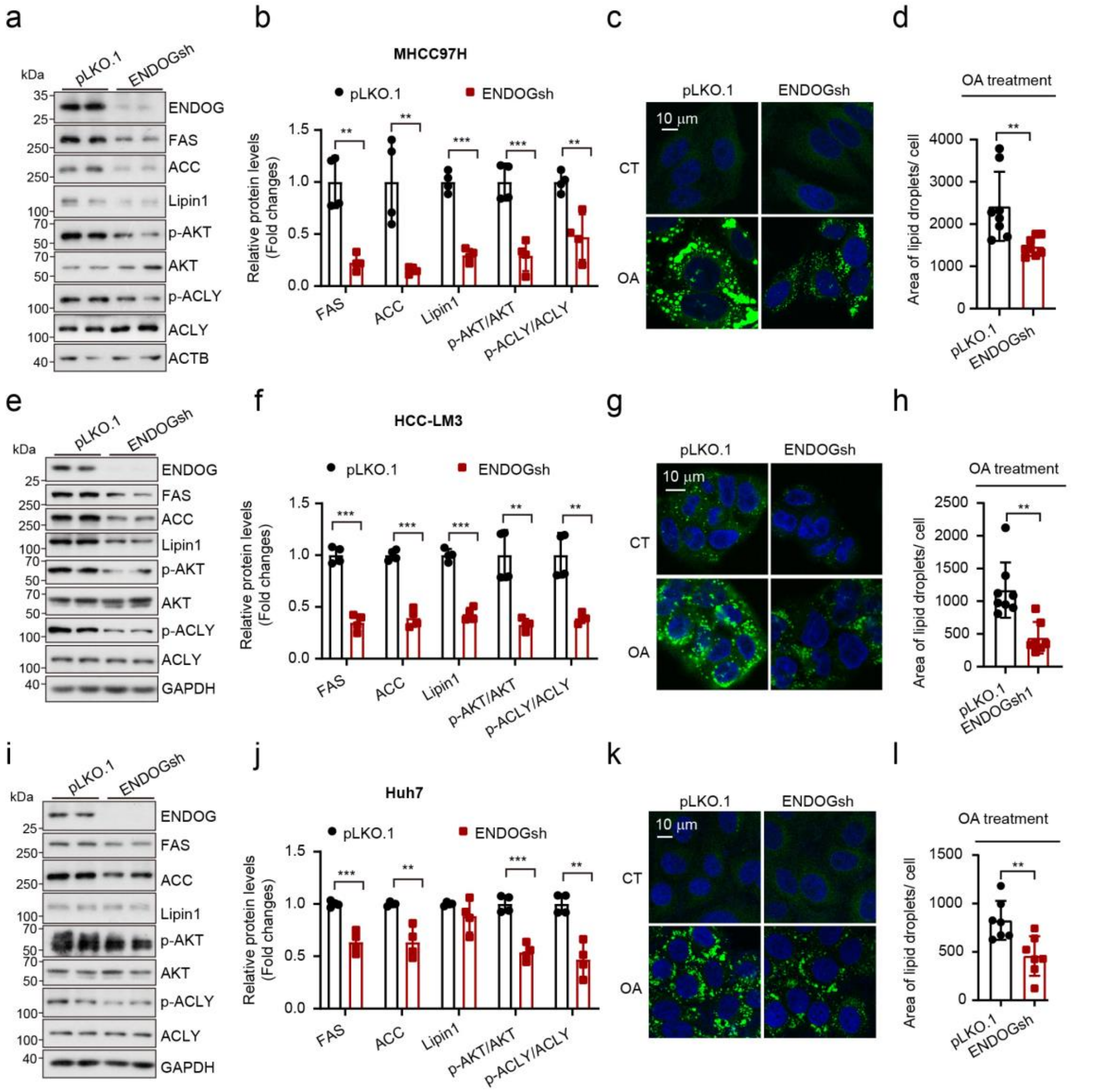

Figure S19. Loss of ENDOG repressed lipid synthesis in hepatocytes. a-b Western bots and quantitative results of lipid synthesis proteins and the AKT-ACLY axis in MHCC97H cells. $n = 4$ each group. **c-d** Representative images of BODIPY in pLKO.1 and ENDOG knockdown MHCC97H cells following the oleic acid treatment. $n = 8$ for each group. **e-f** Western bots and quantitative results of lipid synthesis proteins and the AKT-ACLY axis in HCC-LM3 cells. $n = 4$ for

each group. **g-h** Representative images of BODIPY in pLKO.1 and ENDOG knockdown HCC-LM3 cells following the oleic acid treatment. *n* = 8 for each group. **i-j** Western bots and quantitative results of lipid synthesis proteins and the AKT-ACLY axis in Huh-7 cells. *n* = 4 for each group. **k-l** Representative images of BODIPY in pLKO.1 and ENDOG knockdown Huh-7 cells following the oleic acid treatment. *n* = 8 for each group. pLKO.1: the control cells; ENDOGsh: ENDOG knockdown cells; CT: control; OA: 200 μM oleic acid for 24 hours; Statistical significance was determined by unpaired Student's t-test (two-tailed); error bars are mean ± SD. Source data and exact *P* values are provided in a Source data file. ** $P < 0.01$; *** $P < 0.001$.

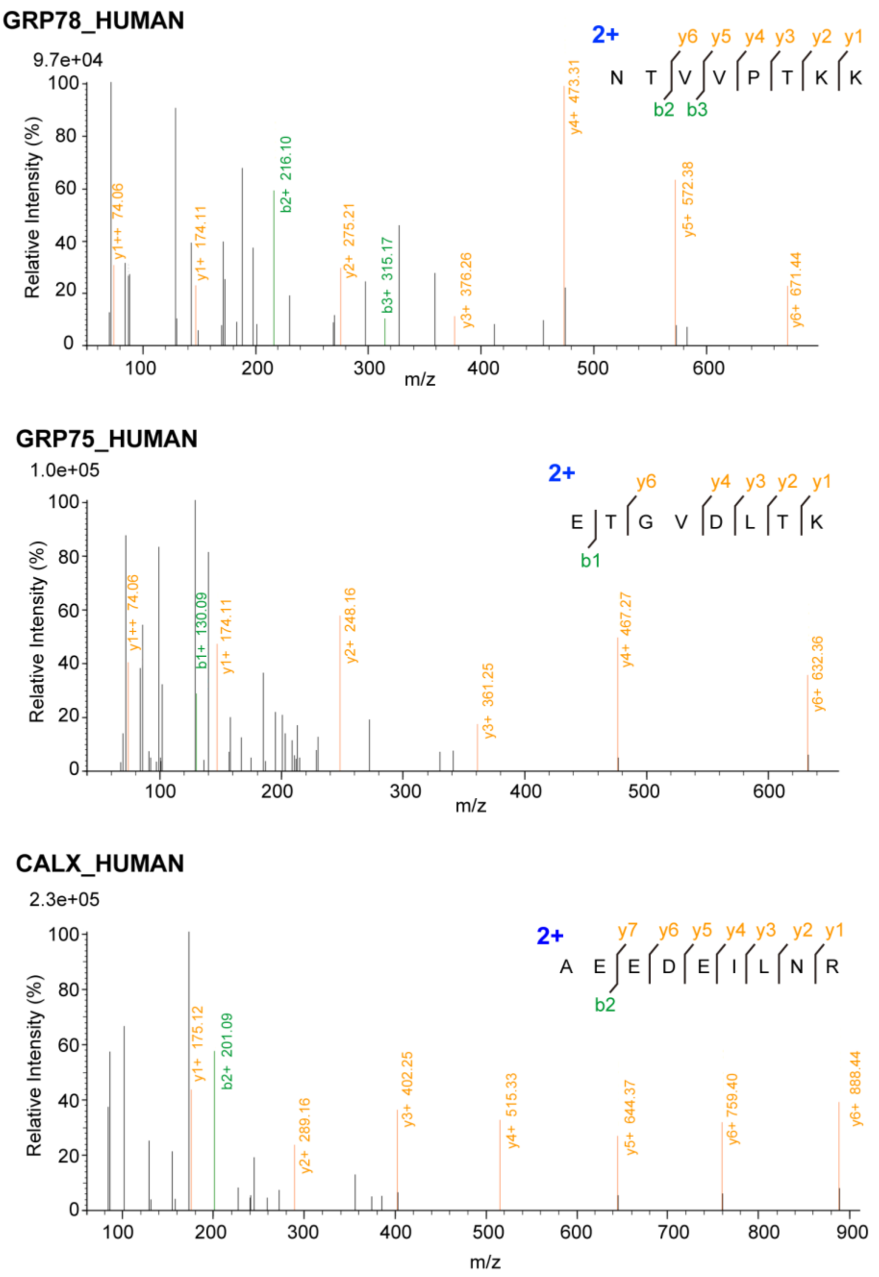

Figure S20. ENDOG interactives with ER resident proteins. Mass spectrometry (MS) analyses of GRP78, GRP75, and Calnexin peptides.

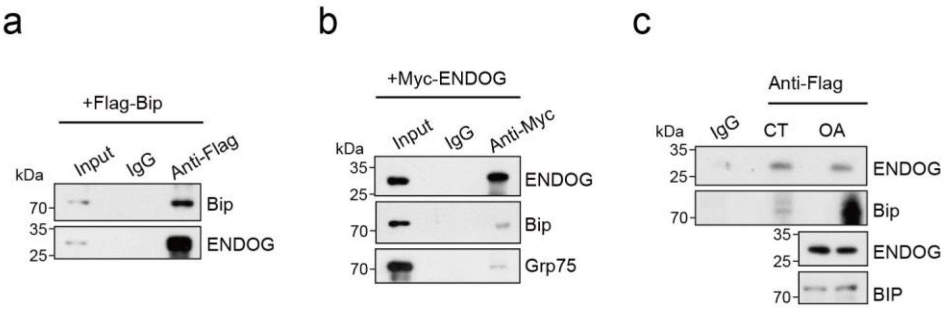

Figure S21. ENDOG bound with Bip and Grp75. a-b Co-IP results in 293T cells. 293T cells were transiently transfected with Flag-Bip or Myc-ENDOG for

48 hours. **c** Co-IP results. 293T cells were transiently transfected with Flag-ENDOG for 24 hours and then treated with or without 200 μM oleic acid for another 24 hours. These data had three independent experiments with similar results.

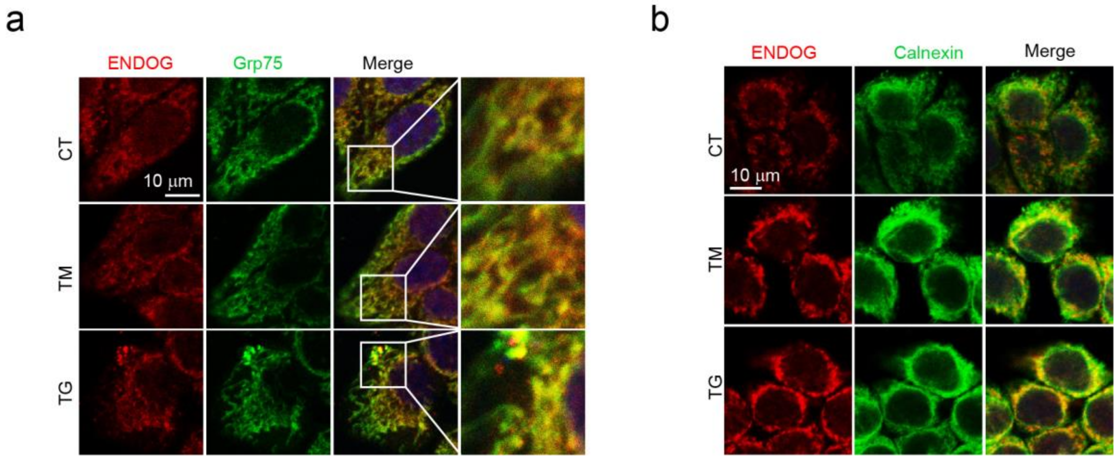

Figure S22. ER stress inductors promote the translocation of ENDOG to the ER. a-b Representative images of costaining of ENDOG and Grp75 or Calnexin. Cells were treated with 2 μg/ml tunicamycin (TM) and 1 μM thapsigargin (TG) for 24 hours.

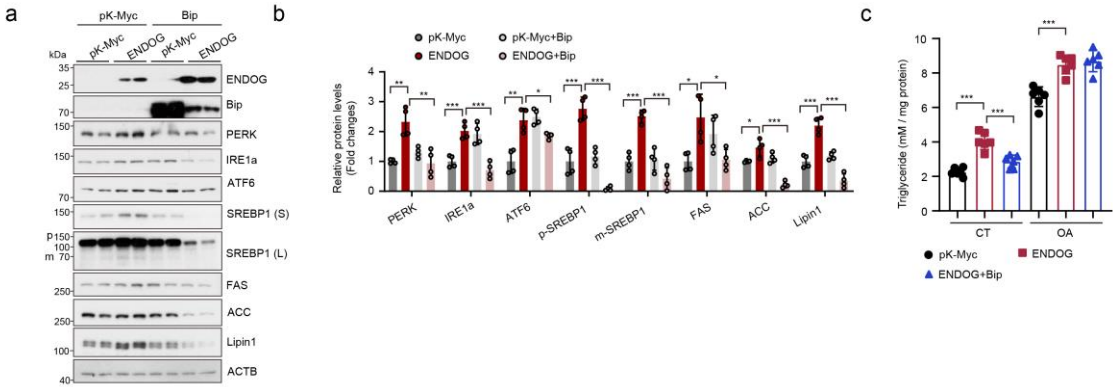

Figure S23. Overexpression of Bip partially represses ENDOG-mediated ER stress and lipid accumulation. a-b Western blot and quantitative results of ER stress-related and lipid synthesis proteins. Cells were transfected with the indicated plasmids for 48 hours. *n* = 4 for each group. p, precursor SREBP1; m, mature SREBP1. **c** Measurement of triglycerides. Cells were transfected with the indicated plasmids for 24 hours and then treated with 200 μM oleic acid for another 24 hours. *n* =6 for each group. Statistical significance was determined by unpaired Student’s t-test (two-tailed); error bars are mean ± SD.

Source data and exact *P* values are provided in a Source data file.* *P* < 0.05; ** *P* < 0.01; *** *P* < 0.001.

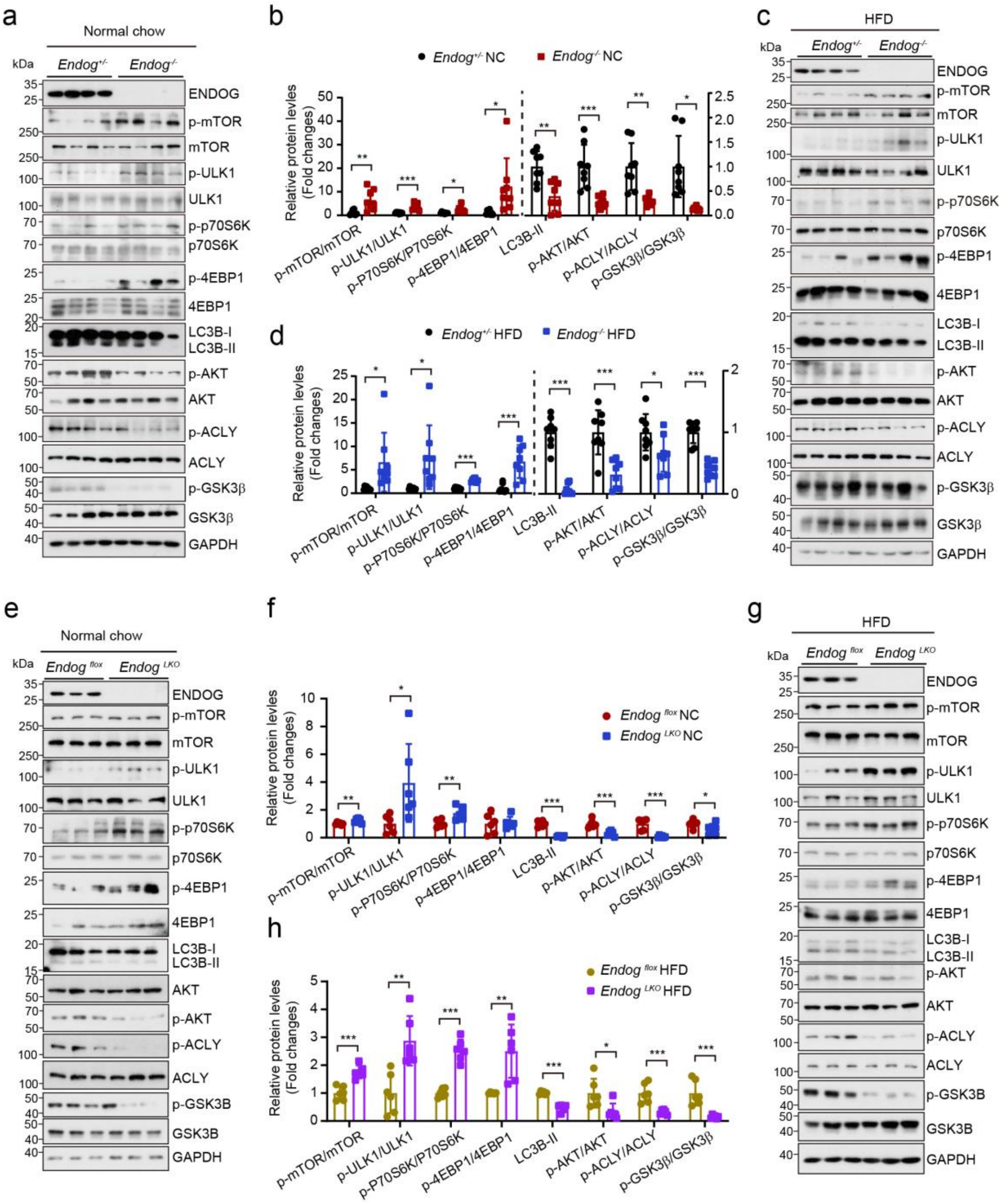

Figure S24. Loss of ENDOG activated mTORC1 and repressed mTORC2 in normal and HFD chow in female mice livers. a-d Representative western blots and quantitative results of the mTORC1 and mTORC2 pathway proteins in Endog$^{+/-}$ / Endog$^{-/-}$ female mice liver under normal and HFD chow. *n* = 8 for each group. **e-g** Representative western blots and quantitative results of the

mTORC1 and mTORC2 pathway proteins in Endog^{flox} / Endog^{LKO} female mice livers under normal and HFD chow. *n* = 6 for each group. NC: normal chow; HFD: high-fat diet chow; Statistical significance was determined by unpaired Student's t-test (two-tailed); error bars are mean ± SD. Source data and exact *P* values are provided in a Source data file.* $P < 0.05$; ** $P < 0.01$; *** $P < 0.001$.

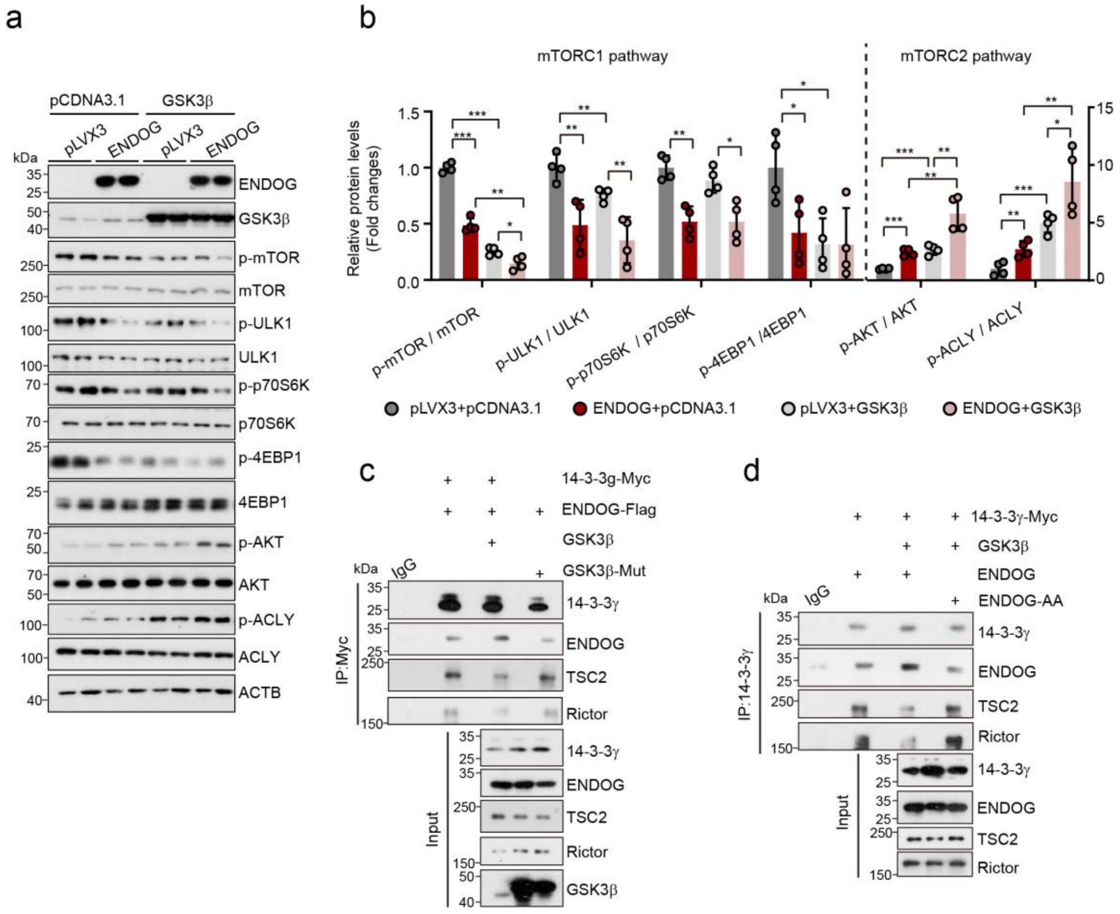

Figure S25 Overexpression of GSK3β enhanced ENDOG-mediated mTORC1 suppression and mTORC2 activation. a-b Western blots and quantitative results of mTORC1 and mTORC2 pathway proteins in pLVX3 and ENDOG overexpressed HepG2 with or without transfected with GSK3β. *n* = 4 for each group. pLVX3: the control cells; ENDOG: ENDOG overexpressed cells; pLVX3 and ENDOG overexpressed HepG2 were transiently transfected with pCNDA3.1 and GSK3β for 48 hours. Statistical significance was determined by unpaired Student's t-test (two-tailed); error bars are mean ± SD. Source data

and exact *P* values are provided in a Source data file.* $P < 0.05$; ** $P < 0.01$; *** $P < 0.001$. **c-d** Co-IP analyses. 293T cells were transiently transfected with the indicated plasmids for 48 hours. ENDOG-AA: ENDOG T128S288 to A128A288. These data had three independent experiments with similar results.

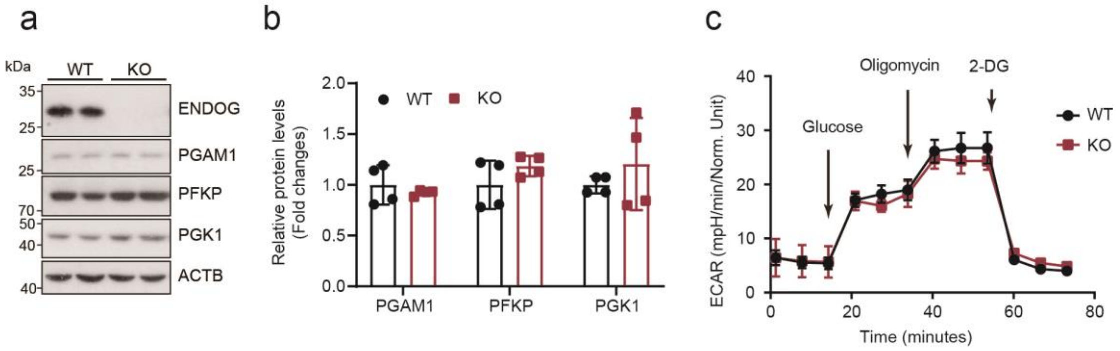

Figure S26. ENDOG has no effects on cell glycolysis. a-b Western blot and quantitative results of glycolysis-related proteins in wild-type and ENDOG knockout cells. *n* = 4 for each group. **c** Seahorse analyses of extracellular acidification rate (ECAR) in wild-type and ENDOG knockout cells (Glucose: 100 mM, Oligomycin: 100 μM, 2-DG: 500 mM). *n* = 5 for each group. Statistical significance was determined by unpaired Student's t-test (two-tailed); error bars are mean ± SD. Source data and exact *P* values are provided in a Source data file.

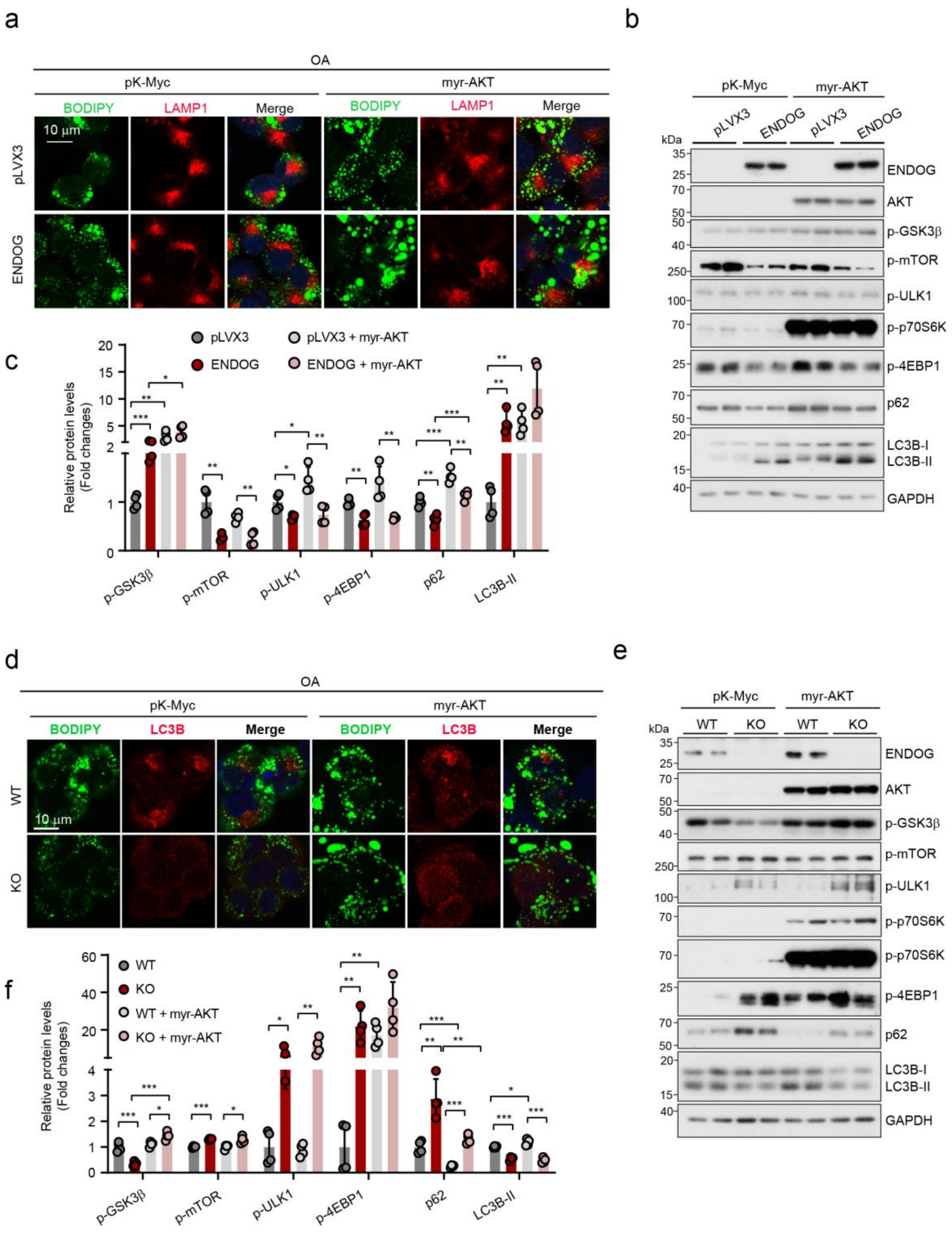

Figure S27. AKT had little influence on ENDOG-mediated mTORC1 suppression and autophagy. a Representative images of BODIPY / LAMP1 co-staining in pLVX3 and ENDOG overexpressed HepG2 cells after transfected with AKT. pLVX3: the control cells; ENDOG: ENDOG overexpressed cells; pLVX3 and ENDOG overexpressed HepG2 were transiently transfected with pK-Myc and myr-AKT for 24 hours and then treated with 200 μM oleic acid for

another 24 hours. **b-c** Western blots and the quantitative results of the indicated proteins. *n* = 4 for each group. pLVX3: the control cells; ENDOG: ENDOG overexpressed cells; pLVX3 and ENDOG overexpressed HepG2 were transiently transfected with pK-Myc and myr-AKT for 48 hours. **d** Representative images of BODIPY / LC3B co-staining in WT and ENDOG knockout HepG2 cells after transfected with AKT. WT: wild-type; KO: ENDOG knockout cells; WT and ENDOG KO HepG2 cells were transiently transfected with pK-Myc and myr-AKT for 24 hours and then treated with 200 μM oleic acid for another 24 hours. **e-f** Western blots and the quantitative results of the indicated proteins. *n* = 4 for each group. WT: wild-type; KO: ENDOG knockout cells; WT and ENDOG KO HepG2 cells were transiently transfected with pK-Myc and myr-AKT for 48 hours. Statistical significance was determined by unpaired Student's t-test (two-tailed); error bars are mean ± SD. Source data and exact *P* values are provided in a Source data file.* $P < 0.05$; ** $P < 0.01$; *** $P < 0.001$.

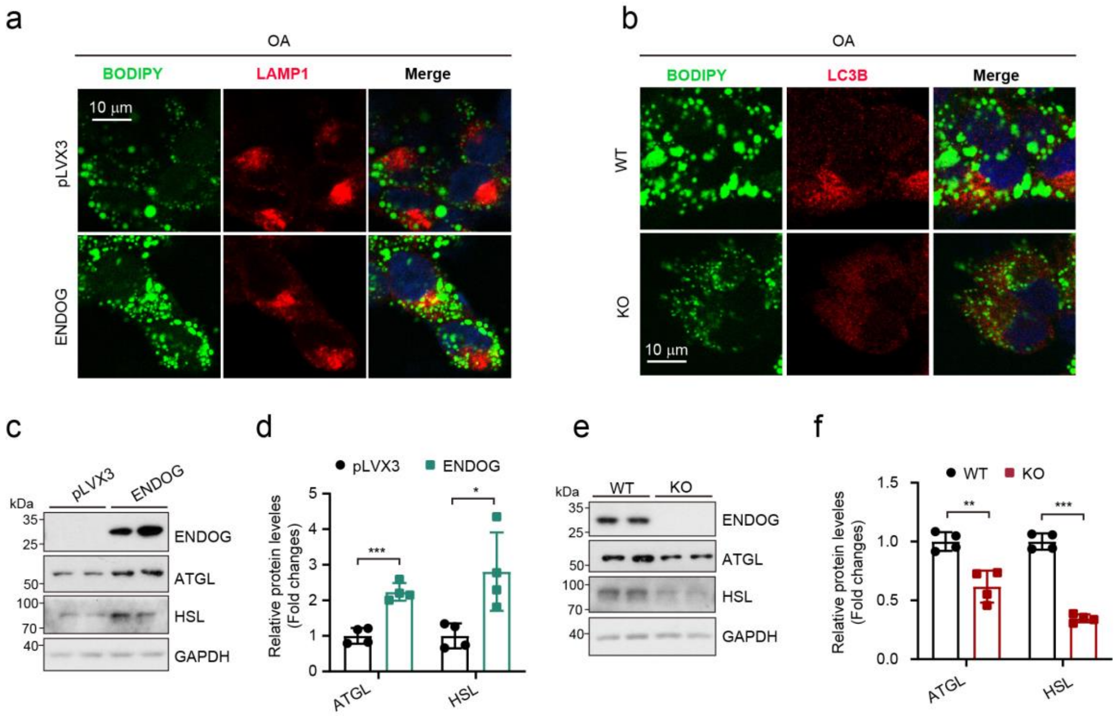

Figure S28. ENDOG promoted the expression of lipolysis. a-b Representative images of BODIPY/LAMP1 and BODIPY / LC3B co-staining in ENDOG overexpressed and knockout HepG2 cells. Cells were treated with 200

μM oleic acid for 24 hours. pLVX3: the control cells; ENDOG: ENDOG overexpressed cells; WT:wild-type; KO: ENDOG knockout. OA: 200 μM oleic acid for 24 hours. **c-f** Western blots and quantitative results of ATGL and HSL in ENDOG overexpressed (c-d) and knockout (e-f) HepG2 cells. *n* = 4 for each group. Statistical significance was determined by unpaired Student's t-test (two-tailed); error bars are mean ± SD. Source data and exact *P* values are provided in a Source data file.* $P < 0.05$; ** $P < 0.01$; *** $P < 0.001$.

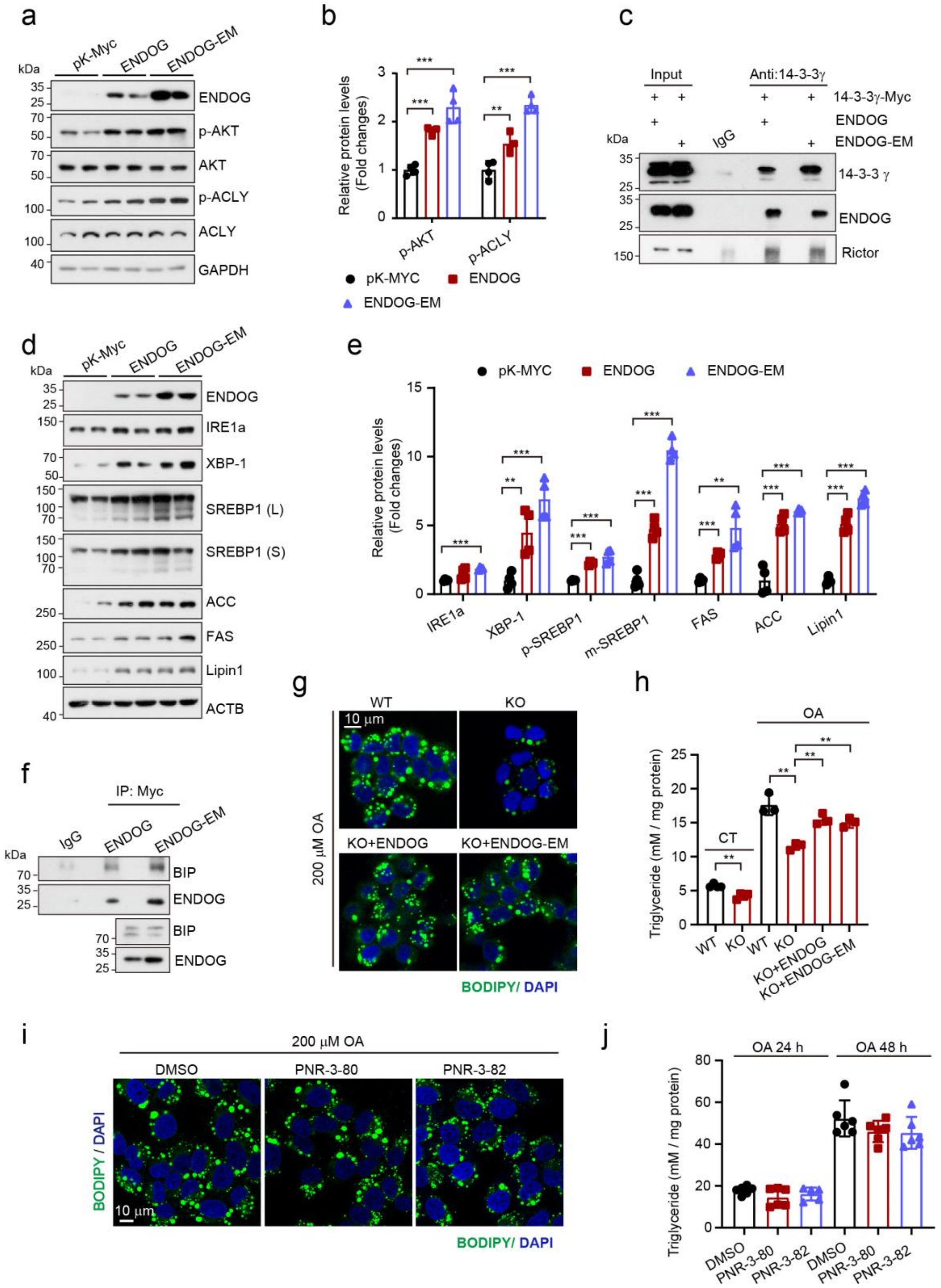

Figure S28. ENDOG-promoted lipid synthesis is endonuclease activity independent. a-b Western blots and quantitative results of AKT-ACLY axis in ENDOG knockout HepG2 cells after transfected with pK-Myc, ENDOG, and ENDOG-EM for 48 hours. *n* = 4 independent samples. **c** Co-IP analyses. 293T cells were cotransfected with 14-3-3γ/ ENDOG or 14-3-3γ/ ENDOG-Mut for 48

hours. **d-e** Western blots and quantitative results of the indicated proteins in ENDOG knockout HepG2 cells after transfected with pK-Myc, ENDOG, and ENDOG-EM for 48 hours. *n* = 4 independent samples. **f** Co-IP analyses. 293T cells were transfected with ENDOG, and ENDOG-EM (Myc tag) for 48 hours. **g-h** Representative images of BODIPY staining and triglyceride measurement (*n* = 4 independent samples). ENDOG knockout HepG2 cells were transfected with pK-Myc, ENDOG, and ENDOG-EM for 24 hours and treated with 200 μM oleic acid for another 24 hours. **i-j** Representative images of BODIPY staining and triglyceride measurement (*n* = 6 independent samples). HepG2 cells were treated with 200 μM oleic acid with or without ENDOG inhibitor (PNR-3-80, PNR-3-82: 50 μM). Statistical significance was determined by unpaired Student's t-test (two-tailed) in **(b, e, h, j);** error bars are mean ± SD. Source data and exact *P* values are provided in a Source data file. ** $P < 0.01$; *** $P < 0.001$.

Table 1. Primary and secondary antibodies used in the present study

Antibody	Company	Catalog number
ACTB	Sigma	Cat: A8481
GAPDH	Cell Signaling Technology	Cat: #2118
ENDOG	Cell Signaling Technology	Cat: #4969
ENDOG	NOVUS	Cat: IMG-5565-2
p-ACLY (Ser455)	Cell Signaling Technology	Cat: #4331T
ACLY	Cell Signaling Technology	Cat: #4332T
FAS	Cell Signaling Technology	Cat: #3180T
ACC	Cell Signaling Technology	Cat: #3676
Lipin1	Cell Signaling Technology	Cat: #14906T
PI3K	Cell Signaling Technology	Cat: #4249
PTEN	Cell Signaling Technology	Cat: #9188
p-PTEN(Ser380)	Cell Signaling Technology	Cat: #9551
p-AKT(Ser473)	Cell Signaling Technology	Cat: #4060
p-AKT(Thr308)	Cell Signaling Technology	Cat: #13038
AKT	Cell Signaling Technology	Cat: #9272
Rictor	Proteintech	Cat: 27248-1-AP
Rictor	Novus	Cat: NB100-612
14-3-3γ	Proteintech	Cat: 12381-1-AP
14-3-3γ	Santa Cruz	Cat: sc-398423
IRE1a	Proteintech	Cat: 27528-1-AP
PERK	Proteintech	Cat: 20582-1-AP
ATF6	Proteintech	Cat: 24169-1-AP
Bip	Proteintech	Cat: 11587-1-AP
Bip	Proteintech	Cat: 66574-1-Ig
CHOP	Proteintech	Cat: 15204-1-AP
XBP-1	Cell Signaling Technology	Cat: # 40435S
EIF2a	Cell Signaling Technology	Cat: #5324

p-EIF2a	Cell Signaling Technology	Cat: #3398
SREBP1	Santa Cruz	Cat: sc-365513
Tim23	Proteintech	Cat: 11123-1-AP
Tim23	Proteintech	Cat: 67535-1-Ig
SDHA	Proteintech	Cat: 14865-1-AP
Cytochrome c	Santa Cruz	Cat:sc-13560
Grp75	Proteintech	Cat:14887-1-AP
PDI	Proteintech	Cat:66422-1-Ig
H3	Cell Signaling Technology	Cat: # 9715
GSK3β	Proteintech	Cat:22104-1-AP
p-GSK3β (Ser 9)	Cell Signaling Technology	Cat:#9323
p62	Sigma	Cat: P0067
LC3B	Sigma	Cat: L7543
LAMP1	Cell Signaling Technology	Cat:#9091
ATGL	Proteintech	Cat:55190-1-AP
HSL	Proteintech	Cat:17333-1-AP
PPARγ	Proteintech	Cat:16643-1-AP
ChREBP1	Proteintech	Cat:13256-1-AP
FABP4	Proteintech	Cat:12802-1-AP
CRTC2	Proteintech	Cat: 12497-1-AP
CREBH (CREB3L3)	NOVUS	Cat: NBP2-16008
SCD1	Proteintech	Cat: 28678-1-AP
DGAT1	Proteintech	Cat: 11561-1-AP
DGAT2	Proteintech	Cat: 17100-1-AP
PLIN2	Proteintech	Cat: 15294-1-AP
CPT1	Abcam	Cat: ab128568
CPT2	Proteintech	Cat: 26555-1-AP
AceCS1	Cell Signaling Technology	Cat: #3658
ALDH2	Proteintech	Cat: 15310-1-AP

mTOR	Cell Signaling Technology	Cat: #2983
p-mTOR	Cell Signaling Technology	Cat: #5536
ULK1	Cell Signaling Technology	Cat: # 8054
p-ULK1	Cell Signaling Technology	Cat: #14202
p70S6K	Cell Signaling Technology	Cat: #2708
p-p70S6K	Cell Signaling Technology	Cat: #9234
4EBP1	Proteintech	Cat: 60246-1-Ig
p-4EBP1	Cell Signaling Technology	Cat: #2855
Mouse Anti-Rabbit IgG (Light-Chain Specific) (D4W3E) mAb (HRP Conjugate)	Cell Signaling Technology	Cat: 93702S
VeriBlot for IP Detection Reagent (HRP)	Abcam	Cat: ab131366
Peroxidase-AffiniPure Goat Anti-Mouse IgG (H+L)	Jackson Immunoresearch	Cat: 115-035-003
Peroxidase-AffiniPure Goat Anti-Rabbit IgG (H+L)	Jackson Immunoresearch	Cat: 111-035-003
Goat anti-Mouse IgG(H+L) Cross-Adsobed Secondary Antibody,Alexa Fluor 488	Thermo	Cat: A28175
Goat anti-Rabbit IgG(H+L) Cross-Adsobed Secondary Antibody,Alexa Fluor 546	Thermo	Cat: A11010
Alexa Fluor® 488-AffiniPure Goat Anti-Rabbit IgG (H+L)	Jackson Immunoresearch	Cat: 111-545-144
Alexa Fluor® 594-AffiniPure Goat Anti-Mouse IgG (H+L)	Jackson Immunoresearch	Cat: 115-585-146

Table 2. Reagents and plasmids used in the present study

**Chemicals or plasmids**	**Company**	**Catalog number**
Oleic acid	Sigma	Cat: L1376
Palmitic acid	Sigma	Cat: 57-10-3
LY294002	Selleck	Cat: S1105

Sodium citrate	Sigma	Cat: 1613859-1G
Acetyl-CoA	Sigma	Cat: A2056-5MG
Insulin	Sigma	Cat: I3536
Tunicamycin	MedChemExpress	Cat: HY-A0098
Thapsigargin	MedChemExpress	Cat: HY- 13433
VBIT-12<br>Digitonin	Selleck<br>Sigma	Cat: S8936<br>Cat: D141-100MG
Mouse High-fat diet	Research Diets Inc	D12492
Mouse normal diet	Beijing HFK Bioscience Co., Ltd.	1032
Triglyceride analysis kit	Applygen Technologies Inc.	Cat: E1013-105
Acetyl CoA Assay Kit	Abcam	Cat: ab87546
Acetyl CoA Assay Kit	Sangon Biotech	Cat: D751001-0096
Free fatty acid kit	Solarbio Life Science	Cat: BC0595
In situ PLA assay	Sigma	Cat: DUO92101
ABScript II cDNA First Strand Synthesis Kit	ABclonal	Cat: RK20400
Mouse INS(Insulin) ELISA Kit	Sangon Biotech	Cat: D721159-0096
Nile red	Sigma	Cat: 72458
BODIPY^{TM} 493/503	Invitrogen	Cat: D3922
Oil red o	Sigma	Cat: O1391
DAPI	Sigma	Cat: D8417
**Plasmids**		
pK-Myc	Addgene	Cat: #19400
pLVX3-ENDOG-Flag	This paper	Expression construct generated in the lab

pK-Myc-ENDOG	This paper	Expression construct generated in the lab
pK-Myc-14-3-3γ	This paper	Expression construct generated in the lab
pLVX3-Bip-Flag	This paper	Expression construct generated in the lab
pCDNA3.1-myr-AKT	Addgene	Cat: #9008
pK-Myc-ENDOG-AA	This paper	Expression construct generated in the lab
pCDNA3.1-GSK3β	Addgene	Cat: 14753
pCDNA3.1-GSK3β-Mut (K85A)	Addgene	Cat: 14755

Table S3. Primers sequence in the present study

Gene:	Sequences: (5'-3')
ACTB-Human	GTTGTCGACGACGAGCG
	GCACAGAGCCTCGCCTT
Endog-Mouse	GTGCCATTGTTGCCGGTG
	AGCTAAGCACGTAGGACTCG
Actb-Mouse	CACTGTCGAGTCGCGTCC
	CGCAGCGATATCGTCATCCA
Pparg-Mouse	TGTGAGACCAACAGCCTGAC
	TCAGTGGTTCACCGCTTCTT
Chrebp1-Mouse	CCAGAGACAACAACCCCTGT
	AAACTGTATCCTGCGGGGAC
Srebp1-Mouse	ACTGGACACAGCGGTTTTGA
	CTGTCTCACCCCCAGCATAG
Srebp2-Mouse	CAAAGGGAGTTGAGCAGCAAG

	TCAGGGAACTCTCCCACTTGA
Fabp4-Mouse	ATCATAACCCTAGATGGCGGG
	CTTTCATAACACATTCCACCAGC
Crtc2-Mouse	CGTCCAATCCACGCAAGTTT
	AGTCAGAGCTTGTCCCGTGT
Crebh-Mouse	AGGTGTAGTGTTTGGGGCTTC
	ACCCGAGCTCCATGTTTCTGTTT
Scd1h-Mouse	GGAAAGTGAGGCGAGCAACT
	TGTAAGAACTGGAGATCTCTTGGA
Dgat1-Mouse	TGACCTCAGCCTTTTCCATGAGT
	CCACACAGCTGCATTGCCATAGTT
Dgat2-Mouse	AACCTGCTGACCACCAGGAACTAT
	AGGGCCTTATGCCAGGAAACTTCT
FASN-Human	TCTCCGACTCTGGCAGCTT
	GCTCCAGCCTCGCTCTC
ACC1-Human	AGTGGGTCACCCCATTGTT
	TTCTAACAGGAGCTGGAGCC
ACC2-Human	CAGCTTCTTGTGTTCCCGTC
	CCTGGAGGCTTATCTGACCA
LPIN1-Human	AAAGGAGAATCCACCAGGAGAC
	ATTCATGGTCTGCACTCTGCT
ACLY-Human	TCCGGATTTTGCGGGGTTC
	GGATGGCTGAGGTGGTACAG
CPT1-Human	GCCTCGTATGTGAGGCAAAA
	TCATCAAGAAATGTCGCACG
CPT-2-Human	CGGAGTCTCGAGCAGATAGG
	GGAAAAGAACTGCATGAGCA
ACOX1-Human	ATGCCCAAGTGAAGATCCAG
	GAAGATGAGGGAGTTTGGCA

FATP2-Human	TCACTTTTTCCACTCCTGCCT
	GTGAGGCCAGTTCCATACCA
FATP5-Human	TGATGGGACTTGTCGTTGGG
	CATTTGCCCGAAGTCCATTGC
CD36-Human	GTGCAAAATCCACAGGAAGTGA
	GACTGTGTTGTCCTCAGCGT
LDLR-Human	AACATGGCTAGAGACTGCCG
	TCATTGCAGACGTGGGAACA
MTTP-Human	CACCTCAGGACTGCGAAGAA
	GGTCTGAGCAGAGGTGACAG
PPARG-Human	CCAGAAGCCTGCATTTCTGC
	TGGCATCTCTGTGTCAACCA
CHREBP1-Human	CCAGACAGCAACAAGACCGA
	GAGCCCATGAAGGGTGTCAA
SREBP1-Human	GCTGCTGACCGACATCGAA
	AGCATGTCTTCGAAAGTGCAATC
SREBP2-Human	AGCTGACCCTGGGAGACATC
	AGCTGTTCTGAAAACAAGTCAGG
CRTC2-Human	GGACCCCAAAGTACCTGCT
	GGCTGGTCAGGAGATGGAAA
CREBH-Human	GCGCGGCTACCTACTTCTTA
	CGAGATCCATGCTTCTTGCC

Table S4. ACLY-shRNA sequence

	Sequence (5'-3')
Oligomer 1:	CCGGGGCATGTCCAACGAGCTCAATCTCGAGATTGA GCTCGTTGGACATGCCTTTTTG
Oligomer 2	AATTCAAAAAGGCATGTCCAACGAGCTCAATCTCGAG ATTGAGCTCGTTGGACATGCC

Table S5. ENDOG-shRNA sequence

	Sequence (5'-3')
Oligomer 1:	GATCCCCAGTCGTACGTGCTGTGCTATTCAAGAGATA GCACAGCACGTAC
Oligomer 2	TCGAATTTTTTCAGCATGCACGACACGATAGAGAACTT ATCGTGTCGTGC
